# Human GBP1 facilitates the rupture of the *Legionella-*containing vacuole and inflammasome activation

**DOI:** 10.1128/mbio.01707-23

**Published:** 2023-09-22

**Authors:** Antonia R. Bass, Marisa S. Egan, Jasmine Alexander-Floyd, Natasha Lopes Fischer, Jessica Doerner, Sunny Shin

**Affiliations:** 1 Department of Microbiology, Perelman School of Medicine, University of Pennsylvania, Philadelphia, Pennsylvania, USA; Yale University School of Medicine, New Haven, Connecticut, USA

**Keywords:** *Legionella pneumophila*, innate immunity, macrophages, inflammasomes, guanylate-binding proteins, caspase-4

## Abstract

The inflammasome is essential for host defense against intracellular bacterial pathogens, including *Legionella pneumophila*, the causative agent of the severe pneumonia Legionnaires’ disease. Inflammasomes recruit and activate caspases, which promote IL-1 family cytokine release and pyroptosis to restrict infection. In mice, interferon (IFN) signaling promotes inflammasome responses against *L. pneumophila* and other bacteria*,* in part, through inducing a family of IFN-inducible GTPases known as guanylate-binding proteins (GBPs). Within murine macrophages, IFN promotes the rupture of the *L. pneumophila*-containing vacuole (LCV), while GBPs are dispensable for vacuole rupture. Instead, GBPs facilitate the lysis of cytosol-exposed *L. pneumophila*. In contrast, the functions of IFN-γ and GBPs in human inflammasome responses to *L. pneumophila* are poorly understood. Here, we show that IFN-γ enhances caspase-1- and caspase-4-dependent inflammasome responses to *L. pneumophila* in human macrophages. We find that human GBP1 is required for these IFN-γ-driven inflammasome responses. Furthermore, we find that GBP1 co-localizes with *L. pneumophila* and/or LCVs in a type IV secretion system (T4SS)-dependent manner and facilitates damage to the LCV, resulting in increased bacterial access to the host cell cytosol. Our findings reveal species- and pathogen-specific differences in how GBPs function during infection.

**Importance:**

Inflammasomes are essential for host defense against intracellular bacterial pathogens like *Legionella*, as they activate caspases, which promote cytokine release and cell death to control infection. In mice, interferon (IFN) signaling promotes inflammasome responses against bacteria by inducing a family of IFN-inducible GTPases known as guanylate-binding proteins (GBPs). Within murine macrophages, IFN promotes the rupture of the *Legionella*-containing vacuole (LCV), while GBPs are dispensable for this process. Instead, GBPs facilitate the lysis of cytosol-exposed *Legionella*. In contrast, the functions of IFN and GBPs in human inflammasome responses to *Legionella* are poorly understood. We show that IFN-γ enhances inflammasome responses to *Legionella* in human macrophages. Human GBP1 is required for these IFN-γ-driven inflammasome responses. Furthermore, GBP1 co-localizes with *Legionella* and/or LCVs in a type IV secretion system (T4SS)-dependent manner and promotes damage to the LCV, which leads to increased exposure of the bacteria to the host cell cytosol. Thus, our findings reveal species- and pathogen-specific differences in how GBPs function to promote inflammasome responses.

## INTRODUCTION

The innate immune response to bacterial pathogens is essential for host defense and bacterial clearance. This response is initiated through the recognition of conserved pathogen-associated molecular patterns by pattern recognition receptors (PRRs) (1). Cytosolic PRRs that detect bacterial components contaminating the host cell cytosol and other virulence activities are critical for host defense against intracellular pathogens. A subset of cytosolic PRRs, the nucleotide-binding oligomerization domain-like receptors, mediate the formation of a multimeric protein complex termed the inflammasome (2). Inflammasome activation culminates in the release of IL-1 family cytokines and an inflammatory form of cell death termed pyroptosis. This response alerts and recruits other innate immune cells to the site of infection, thereby promoting bacterial clearance.

Two types of inflammasomes have been described: the canonical and noncanonical inflammasomes. In response to a diverse range of signals, canonical inflammasomes activate the cysteine protease caspase-1 to promote the processing and release of the proinflammatory cytokines IL-1β and IL-18 (2, 3). An alternative caspase-1-independent inflammasome, termed the noncanonical inflammasome, mediates inflammatory responses to Gram-negative bacteria (4 – 9). The noncanonical inflammasome is formed by caspase-11 in mice and two orthologs in humans, caspase-4 and caspase-5. These caspases are activated upon binding of lipopolysaccharide (LPS), a major outer membrane component of Gram-negative bacteria (10 – 12). Following their activation, the inflammatory caspases cleave the substrate gasdermin-D (GSDMD). The liberated GSDMD N-terminal fragment translocates to the plasma membrane and oligomerizes to form a pore, leading to IL-1 cytokine release and pyroptosis (13, 14). Death of the infected cell eliminates the replicative niche for intracellular pathogens and leads to the clearance of bacteria through various mechanisms, including efferocytosis of bacteria within pore-induced intracellular traps by neutrophils (15).

Inflammasome responses are potentiated by priming signals, such as Toll-like receptors and type I and type II IFN signaling, that upregulate expression of inflammasome-related components. A subfamily of IFN-induced GTPases called guanylate-binding proteins (GBPs) are particularly important in promoting inflammasome responses to Gram-negative bacteria in mice (16 – 24). Murine GBPs can localize to pathogen-containing vacuoles or cytosol-exposed bacteria (17, 21, 25). The precise steps regulated by murine GBPs in promoting inflammasome activation are unclear. Earlier studies with *Salmonella* Typhimurium indicated that murine GBPs promote the rupture of pathogen-containing vacuoles (PCVs), whereas other studies with *Francisella novicida* and *Legionella pneumophila* indicate that GBPs instead facilitate bacteriolysis, resulting in cytosolic release of bacterial components that trigger inflammasome activation (18, 20, 21, 25, 26). Additionally, murine GBPs promote caspase-11 activation in response to transfected LPS, therefore revealing that GBPs can operate downstream of vacuolar and outer membrane lysis (22). Murine GBPs can also promote inflammasome responses without targeting the PCV, as is the case with *Chlamydia muridarum* (27). It is still unclear how murine GBPs mediate these various functions, although one study showed that murine GBPs recruit the immunity-related GTPase (IRG) IRGB10 to mediate bacteriolysis (25).

While studies in mice have linked the functions of IFN signaling and GBPs to inflammasome activation, the degree to which the function of murine GBPs mirror their human counterparts is unknown. The evolutionary differences in immune genes between mice and humans, including in the GBP superfamily, could translate into differences in immune mechanisms. Notably, mice have 11 GBPs, whereas humans only have seven (28). Understanding the functions of human GBPs in host defense against pathogens, particularly whether they play a role in PCV rupture or bacteriolysis, is incomplete. Human GBP1 directly binds to the LPS of the cytosolic pathogen *Shigella flexneri* and further recruits additional GBPs, specifically GBP2, 3, 4, and 6, to inhibit *S. flexneri’s* actin-based motility (29, 30). Human GBP1 and GBP2 can coat the cytosolic pathogen *Francisella novicida* (31). Additionally, human GBP1 and GBP5 promote inflammasome responses to *S*. Typhimurium, which can reside within a vacuole or the cytosol, while human GBP2 promotes inflammasome responses to the cytosolic pathogen *F. novicida* (23, 32, 33). Intriguingly, human GBP1 also mediates host defense against the eukaryotic pathogen *Toxoplasma gondii*, which lacks LPS, by promoting the rupture of the PCV and parasite membranes (34). Furthermore, human GBP1 associates with ruptured host vacuoles in the absence of infection (29, 35), indicating that human GBPs may be capable of associating with host-derived bacteria-containing vacuoles, similar to mouse GBPs. These findings indicate that the functions of human GBPs vary in a pathogen- and context-specific manner.

Here, we sought to define the role of IFN-γ and human GBPs in human inflammasome responses to *L. pneumophila. L. pneumophila* is a Gram-negative pathogen that is the causative agent of the severe pneumonia Legionnaires’ disease (36, 37). Upon uptake into macrophages, this pathogen relies on the Dot/Icm type IV secretion system (T4SS) to replicate within an *L. pneumophila*-containing vacuole (LCV) (38 – 41). The T4SS injects over 300 effector proteins, many of which enable *L. pneumophila* to evade the endolysosomal pathway and modify its LCV into an ER-derived replicative compartment (42 – 45). T4SS activity also inadvertently activates canonical and noncanonical inflammasomes in human macrophages (46). A recent study found that recombinant human GBP1 can directly bind to the cell membrane of *L. pneumophila* and other Gram-negative bacterial pathogens (47). However, the role of GBP1 and IFN-γ signaling in regulating inflammasome responses to *L. pneumophila* in human macrophages is unclear.

In this study, we found that IFN-γ and human GBP1 promote caspase-1- and caspase-4-dependent inflammasome responses to *L. pneumophila* in both immortalized and primary human macrophages. In IFN-γ-primed macrophages, there was a significant increase in GBP1 and GBP2 recruitment to the LCV and/or outer membrane of *L. pneumophila* in a T4SS-dependent manner. Intriguingly, IFN-γ and GBP1 facilitated the rupture of LCVs, as assessed by galectin-8 recruitment to the LCV and increased exposure of *L. pneumophila* to the host cell cytosol. Overall, our findings indicate that IFN-γ and human GBP1 facilitate LCV rupture, thereby promoting cytosolic exposure of the bacteria to enhance caspase-1- and caspase-4-dependent inflammasome activation.

## RESULTS

### IFN-γ promotes inflammasome responses to *L. pneumophila* in human macrophages

**Fig 1:**
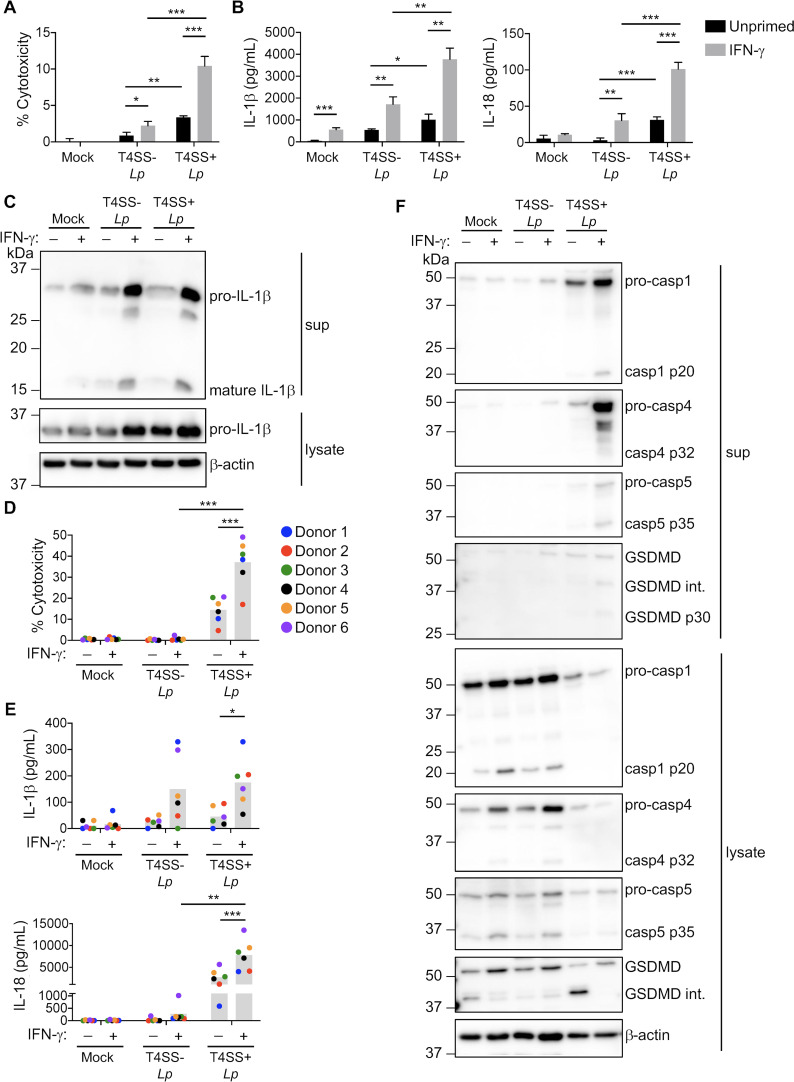
IFN-γ promotes inflammasome responses to *L. pneumophila* in human macrophages. PMA-differentiated THP-1 cells (**A–C**) or primary human monocyte-derived macrophages (hMDMs) (**D–F**) were unprimed or primed with IFN-γ (100 U/mL) overnight and infected with T4SS− *Lp*, T4SS+ *Lp*, or mock-infected with PBS for 2 h (THP-1 cells) or 4 h (hMDMs). (**A, D**) Cell death was measured using LDH release assay and normalized to mock-infected cells. (**B, E**) IL-1β and IL-18 levels in the supernatant were measured by ELISA. (**C, F**) Immunoblot analysis was conducted on supernatants (sup) and lysates from THP-1 cells (**C**) or hMDMs (**F**) for IL-1β, caspase-1, caspase-4, caspase-5, GSDMD, and β-actin. Representative of three independent experiments. (**A, B**) Representative of three independent experiments. *P < 0.05, **P < 0.01, and ***P < 0.001 by unpaired *t*-test. (**D, E**) Shown are the pooled results of six independent experiments using hMDMs from different healthy human donors. Each data point represents the mean of triplicate wells from an individual donor. *P < 0.05, **P < 0.01, and ***P < 0.001 by paired *t*-test.

We first determined whether IFN-γ priming enhances inflammasome responses to *L. pneumophila* in human macrophages and whether these responses require the T4SS. Unprimed or IFN-γ-primed THP-1 macrophages were infected with a *L. pneumophila dotA* mutant lacking a functional T4SS (T4SS−) or a T4SS-sufficient (T4SS+) strain lacking flagellin (Δ*flaA*) in order to focus on NAIP-independent inflammasome responses (48). Unprimed THP-1 cells infected with T4SS− *Lp* or mock-infected exhibited little to no cell death, whereas cells infected with T4SS+ *Lp* underwent increased cell death and IL-1 family cytokine release (Fig. 1A and B), consistent with previous findings (46). With IFN-γ priming, THP-1 macrophages infected with T4SS+ *Lp* underwent significantly increased cell death and IL-1β and IL-18 release compared to unprimed macrophages (Fig. 1A and B; Fig. S1A). Interestingly, we noticed significantly increased release of IL-1β and IL-18 in T4SS− *Lp*-infected THP-1 cells primed with IFN-γ compared to unprimed cells, indicating that IFN-γ potentiates inflammasome responses to *L. pneumophila* lacking a T4SS. Furthermore, IL-1β was processed into its mature p17 form in both T4SS− and T4SS+ *Lp*-infected THP-1 cells primed with IFN-γ (Fig. 1C). However, unprimed and IFN-γ-primed THP-1 cells infected with T4SS− *Lp* released significantly lower levels of IL-1 family cytokines compared to their T4SS+ *Lp*-infected counterparts. These data indicate that IFN-γ priming promotes inflammasome responses to both T4SS− and T4SS+ *L. pneumophila* in THP-1 cells although maximal inflammasome responses are mounted against bacteria harboring a functional T4SS.

We next asked whether IFN-γ enhances inflammasome responses to *L. pneumophila* in primary human monocyte-derived macrophages (hMDMs) derived from healthy human donors. IFN-γ-primed hMDMs infected with T4SS+ *Lp* exhibited significantly increased cell death (Fig. 1D) and IL-1β and IL-18 release (Fig. 1E), compared to unprimed or IFN-γ-primed hMDMs that were infected with T4SS− *Lp* or uninfected. Overall, our data indicate that IFN-γ promotes inflammasome responses to *L. pneumophila* in both THP-1 macrophages and primary hMDMs.

### IFN-γ promotes caspase-1- and caspase-4-dependent inflammasome responses to *L. pneumophila*

We next investigated which caspases are involved in IFN-γ-enhanced inflammasome responses to *L. pneumophila*. We observed caspase-1 processing into its mature p20 form in IFN-γ-primed hMDMs infected with T4SS+ *Lp* (Fig. 1F). Both caspase-4 and caspase-5 were upregulated at the RNA and protein level following IFN-γ priming of THP-1 cells and hMDMs (Fig. 1F; Fig. S1B through D). Additionally, we observed release of full-length and processed forms of caspase-4, caspase-5, and GSDMD into the supernatants of IFN-γ-primed hMDMs infected with T4SS+ *Lp* (Fig. 1F). Unprimed, mock-infected primary hMDMs had basal caspase-5 expression, and IFN-γ priming led to processed caspase-5 p35 subunit in primary hMDMs and THP-1 cells, suggesting that caspase-5 may be constitutively expressed and undergoes some autoprocessing regardless of infection status (Fig. 1F). Together, these data indicate that caspase-1, caspase-4, and caspase-5 are processed into their mature forms upon IFN-γ priming and infection with T4SS+ *Lp* (Fig. 1F).

**Fig 2:**
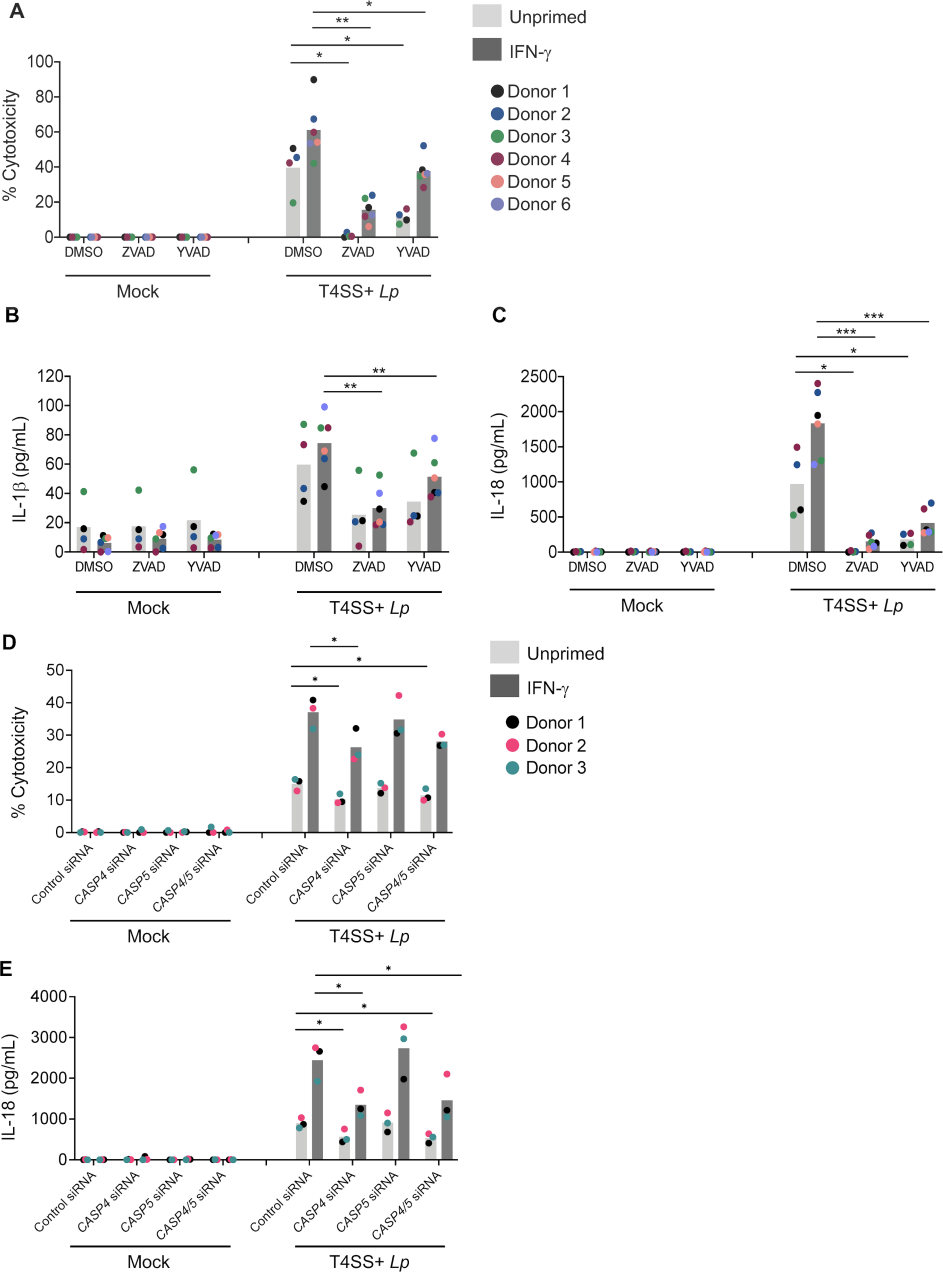
Caspase-4 and caspase activity are required for IFN-γ-primed inflammasome responses to *L. pneumophila*. (**A–C**) Primary hMDMs were unprimed or primed with IFN-γ (100 U/mL) overnight and treated with YVAD, ZVAD, or DMSO vehicle control for 1 h followed by infection with T4SS+ *Lp* for 4 h. (**D, E**) Primary hMDMs were transfected with 30 nM of scrambled control siRNA (siControl), or siRNAs targeting *CASP4* (siCASP4), *CASP5* (siCASP5), or both (siCASP4/5), unprimed or primed with IFN-γ (100 U/mL) overnight, and infected with T4SS+ *Lp* or PBS (Mock) for 4 h. (**A, D**) Cell death was measured using the LDH assay and normalized to mock-infected cells. (**B, C, E**) IL-1β and IL-18 levels in the supernatant were measured by ELISA. (**A, B, C**) Shown are the pooled results of four to six independent experiments using hMDMs from different healthy human donors. Each data point represents the mean of triplicate infected wells from an individual donor. *P < 0.05, **P < 0.01, and ***P < 0.001 by paired *t*-test. (**D, E**) Shown are the pooled results of three independent experiments using hMDMs from three different healthy human donors. Each data point represents the mean of triplicate infected wells from an individual donor. *P < 0.05 by paired *t*-test.

We next asked whether caspase activity is required for inflammasome responses. Cell death and IL-1β and IL-18 release were significantly decreased in either unprimed or IFN-γ-primed hMDMs treated with the pan-caspase inhibitor ZVAD prior to infection with T4SS+ *Lp*, compared to vehicle control-treated cells (Fig. 2A through C). Treatment with the caspase-1-specific inhibitor YVAD also significantly reduced cell death and IL-1β and IL-18 release in IFN-γ-primed or unprimed hMDMs, compared to DMSO-treated hMDMs (Fig. 2A through C), although not decreased to the same extent observed with ZVAD treatment. These data indicate that caspase-1 and additional caspases are involved in inflammasome responses to *L. pneumophila*.

As both caspase-4 and caspase-5 are processed in IFN-γ-primed T4SS+ *Lp*-infected hMDMs (Fig. 1F), we next sought to determine whether these caspases play a role in IFN-γ-mediated inflammasome responses to *L. pneumophilia*. Using siRNAs targeting *CASP4* and *CASP5,* we observed specific knockdown of each caspase (Fig. S2A and B). Cell death and IL-18 release were significantly reduced in unprimed and IFN-γ-primed hMDMs treated with *CASP4* or both *CASP4* and *CASP5* siRNA prior to infection with T4SS+ *Lp*, compared to control siRNA-treated cells (Fig. 2D and E). Knockdown of *CASP4* alone led to a similar decrease in cell death and IL-18 release compared to knockdown of both *CASP4* and *CASP5*. These results suggest that caspase-4 is the primary noncanonical caspase responding to *L. pneumophila*, while caspase-5 is not required. Collectively, our data indicate that IFN-γ priming promotes both caspase-1- and caspase-4-dependent inflammasome responses to *L. pneumophila* in hMDMs.

### Human GBP1 contributes to maximal IFN-γ-dependent inflammasome responses to *L. pneumophila*

In mice, two IFN-inducible gene families that promote inflammasome activation are the GBPs and IRGs. Their assigned functions include binding and rupturing the phagosome of vacuolar pathogens, as well as directly lysing bacteria that escape the phagosome and enter the cytosol (18, 20, 21, 25, 26). Mice have 11 GBPs and 23 IRGs, whereas humans have 7 GBPs and 2 IRG genes (49). Human GBPs, like their murine counterparts, are IFN-inducible, whereas human IRGs are not induced by IFN stimulation (33, 49, 50).

Thus, we tested whether human GBPs play a role in IFN-γ-enhanced inflammasome responses to *L. pneumophila*. In THP-1 cells and hMDMs, expression of most GBPs were induced by IFN-γ (Fig. S3A and B). We observed high relative expression of *GBP1, GBP2, GBP3, GBP4,* and *GBP5,* whereas there was very low relative expression of *GBP6* and *GBP7* in IFN-γ-primed THP-1 cells (Fig. S4A) and hMDMs (Fig. S4B) in agreement with previous findings (33). Furthermore, priming hMDMs with increasing amounts of IFN-γ led to a dose-dependent increase in GBP mRNA and protein levels (Fig. S3C, D and S4C). Thus, GBP expression is induced by IFN-γ in human macrophages, in agreement with previous findings (33, 50).

**Fig 3:**
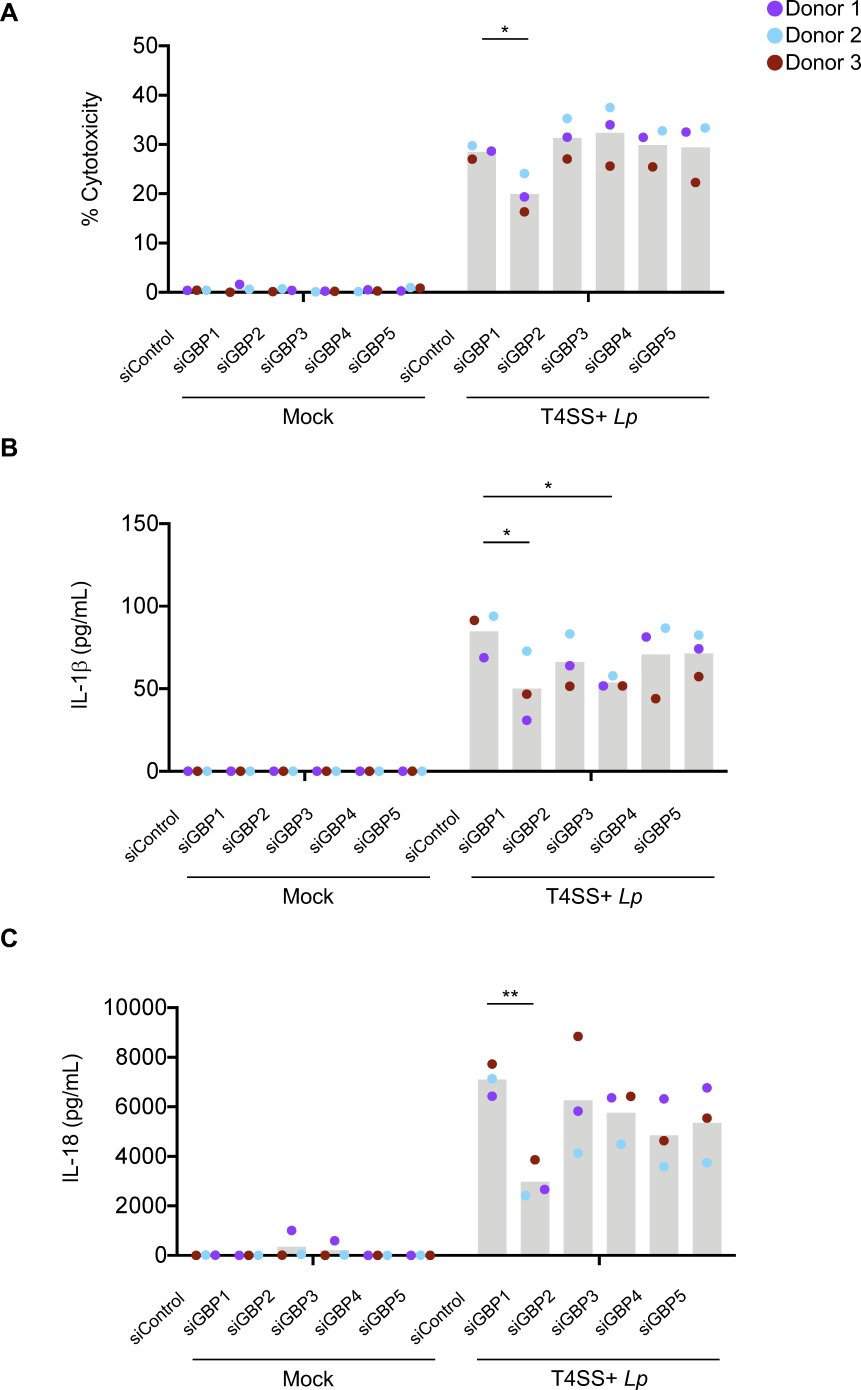
GBP1 is required for maximal IFN-γ-primed inflammasome responses to *L. pneumophila* in primary human macrophages. (**A–C**) Primary hMDMs were transfected with 30 nM siRNA specific for each GBP or scrambled control siRNA (siControl), primed with IFN-γ (100 U/mL) overnight, and infected with T4SS+ *Lp* or PBS (Mock) for 4 h. (**A**) Cell death was measured using the LDH release assay and normalized to mock-infected cells. (**B**) IL-1β and (**C**) IL-18 levels in the supernatant were measured by ELISA. (**A, B, C**) Shown are the pooled results of three independent experiments using hMDMs from different healthy human donors. Each data point represents the mean of triplicate infected wells from an individual donor. *P < 0.05 and **P < 0.01 by paired *t*-test.

Since GBP1–5 were significantly upregulated in IFN-γ-primed hMDMs, we next tested whether these GBPs play a role in IFN-γ-primed inflammasome responses to *L. pneumophila*. We used siRNAs to individually silence expression of *GBP1–5* in hMDMs prior to IFN-γ treatment and T4SS+ *Lp* infection. Notably, knockdown of *GBP1* significantly decreased cell death and IL-1β and IL-18 release following *L. pneumophila* infection in IFN-γ-primed hMDMs, indicating that GBP1 plays a nonredundant role in inflammasome responses against *L. pneumophila* (Fig. 3A through C). *GBP3* knockdown resulted in significantly decreased IL-1β release but did not affect cell death or IL-18 release. In contrast, knockdown with siRNAs against *GBP2, 4,* or *5* did not decrease cell death or cytokine secretion. siRNA knockdown was specific for each GBP and did not affect expression of the remaining GBPs (Fig. S5A through E).

**Fig 4:**
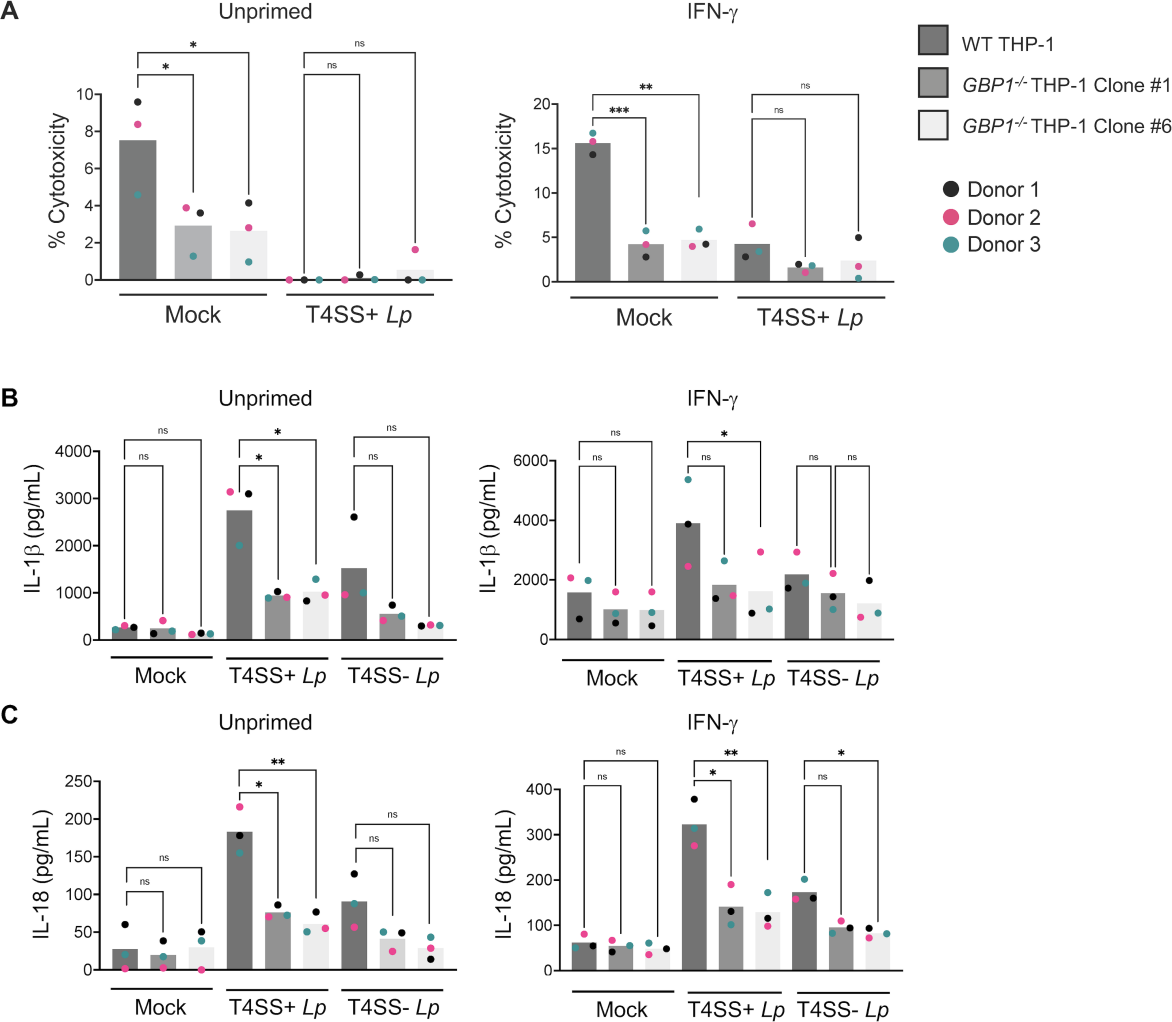
GBP1 is required for IFN-γ-primed inflammasome responses to *L. pneumophila* in THP-1 cells. (**A–C**) PMA-differentiated WT THP-1 cells and *GBP1^−/^* ^−^ clones were either primed with IFN-γ (100 U/mL) overnight or unprimed and infected with T4SS+ *Lp,* T4SS− *Lp,* or PBS (Mock) for 2 h. (**A**) Cell death was measured using the LDH release assay and normalized to mock-infected cells. (**B**) IL-1β and (**C**) IL-18 levels in the supernatant were measured by ELISA. (**A–C**) Shown are the pooled results of three independent experiments. Each data point represents the mean of triplicate infected wells from an individual experiment. Not significant (ns) *P* > 0.05, *P < 0.05, **P < 0.01, ***P < 0.001 by paired *t*-test.

To further validate the role of GBP1 in inflammasome responses to *L. pneumophila,* we used CRISPR-Cas9 technology to generate two sequence-validated human GBP1-deficient (*GBP1^−/^* ^−^) THP-1 single-cell clones that resulted in premature stop codons (Fig. S6A through C). We confirmed by immunoblot analysis that these two *GBP1^−/^* ^−^ THP-1 clones do not express GBP1 when primed with IFN-γ (Fig. S6D). We observed a significant decrease in cell death and IL-1β and IL-18 release in both unprimed and IFN-γ-primed *GBP1^−/−^* THP-1 clones compared to WT THP-1 cells following infection with T4SS+ *Lp*, but not with T4SS− *Lp* (Fig. 4A through C). Collectively, these data indicate that human GBP1 is required for IFN-γ-primed inflammasome responses in both hMDMs and THP-1 cells during infection with T4SS+ *Lp*.

### IFN-γ promotes GBP1 and 2 colocalization with *L. pneumophila* in a T4SS-dependent manner

**Fig 5:**
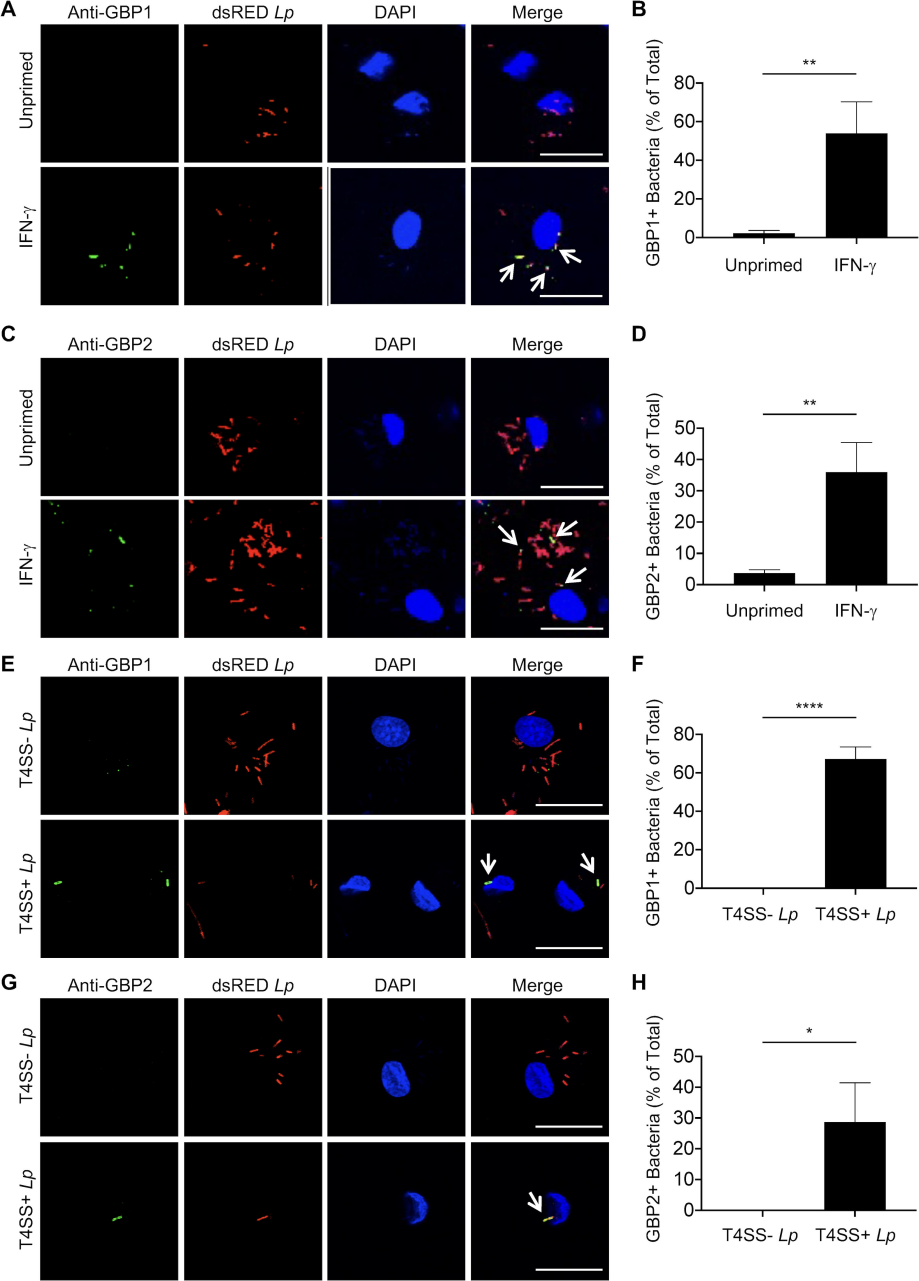
IFN-γ promotes GBP1 and GBP2 colocalization with *L. pneumophila* in a T4SS-dependent manner. (**A–D**) Primary hMDMs were either unprimed or primed with IFN-γ (100 U/mL) overnight and then infected with dsRED-expressing T4SS+ *Lp* for 2 h. (**E–H**) Primary hMDMs were primed with IFN-γ (100 U/mL) overnight and then infected with dsRED-expressing T4SS− *Lp* or T4SS+ *Lp* for 2 h. (**A–D**) Representative fluorescence micrographs of cells stained for GBP1 (**A**) or GBP2 (**C**). Percentage of hMDMs containing GBP1+ *Lp* (**B**) or GBP2+ *Lp* (**D**) out of total infected hMDMs. Graphs show the mean and s.d. of technical triplicates and are representative of three independent experiments using hMDMs from different healthy human donors. **P < 0.01 by unpaired *t*-test. (**E–H**) Representative fluorescence micrographs of cells stained for GBP1 (**E**) or GBP2 (**G**). Quantification of the percentage of hMDMs containing GBP1+ *Lp* (**F**) or GBP2+ *Lp* (**H**) out of total infected hMDMs. Graphs show the mean and s.d. of technical triplicates and are representative of two independent experiments using hMDMs from different healthy human donors. (**A, C, E, G**) Scale bars, 20 µM. White arrows indicate anti-GBP1- or anti-GBP2-stained *L. pneumophila*. *P < 0.05 and ****P < 0.0001 by unpaired *t*-test.

We next asked how GBP1 could be promoting inflammasome responses to *L. pneumophila*. Murine Gbp2 colocalizes with *S*. Typhimurium and promotes the rupture of the *Salmonella*-containing vacuole (SCV) (20), while its predicted human ortholog, GBP1, targets and binds the outer membrane of *S. flexneri* and *S*. Typhimurium to form a GBP complex and disrupt bacterial membrane integrity for recruitment and activation of caspase-4 (32, 34, 47, 51, 52). In addition, recombinant human GBP1 can directly bind to *L. pneumophila* (47). Thus, we hypothesized that human GBP1 would colocalize with the LCV and/or *L. pneumophila* in IFN-γ-primed macrophages. To test this hypothesis, we infected IFN-γ-primed and unprimed hMDMs with dsRED-expressing T4SS+ *Lp* and subsequently stained for GBP1. While there was little to no GBP1 expression in unprimed cells, there was a significant increase in the percentage of infected cells containing GBP1-positive *L. pneumophila* following IFN-γ priming (Fig. 5A and B). Furthermore, approximately 60% of infected cells contained *L. pneumophila* that colocalized with GBP1. In contrast, GBP1 was distributed throughout the cytoplasm in uninfected IFN-γ-primed hMDMs (Fig. S7A). The secondary antibodies used for anti-GBP1 staining did not stain *L. pneumophila* and/or the LCV when used alone (Fig. S7B). Overall, these data indicate that GBP1 is recruited to *L. pneumophila* and/or the LCV within IFN-γ-primed hMDMs.

During infection with the cytosolic bacterium *S. flexneri*, in addition to GBP1, other GBPs are recruited to inhibit bacterial actin-based motility (29, 30). Thus, we tested whether GBP2 also colocalized with *L. pneumophila* and/or the LCV. We observed a significantly increased percentage of hMDMs harboring GBP2+ *L. pneumophila* following IFN-γ priming compared to unprimed cells (Fig. 5C and D) although to a lower extent compared to GBP1+ *L. pneumophila*. Furthermore, secondary antibodies used for anti-GBP2 staining did not stain *L. pneumophila* and/or the LCV when used alone (Fig. S7C). These findings indicate that both GBP1 and GBP2 are recruited to *L. pneumophila* and/or the LCV in IFN-γ-primed hMDMs.

In murine macrophages, colocalization of GBPs with *L. pneumophila* is dependent on the T4SS, and similarly, GBP colocalization with *Yersinia pseudotuberculosis* requires the presence of type III secretion system translocon components (24, 35). These findings indicate that murine GBPs respond to the presence or activity of secretion systems that are key signatures of bacterial virulence or their secreted substrates. However, whether human GBPs also detect PCVs that contain bacteria expressing virulence-associated secretion systems is unclear. Notably, only T4SS+ *Lp*, but not T4SS− *Lp,* exhibited robust colocalization with GBP1 and GBP2 in IFN-γ-primed hMDMs (Fig. 5E through H). Collectively, these data indicate that GBP1 and GBP2 are recruited to *L. pneumophila* and/or the LCV in a T4SS-dependent manner.

### IFN-γ and GBP1 promote the rupture of LCVs

**Fig 6:**
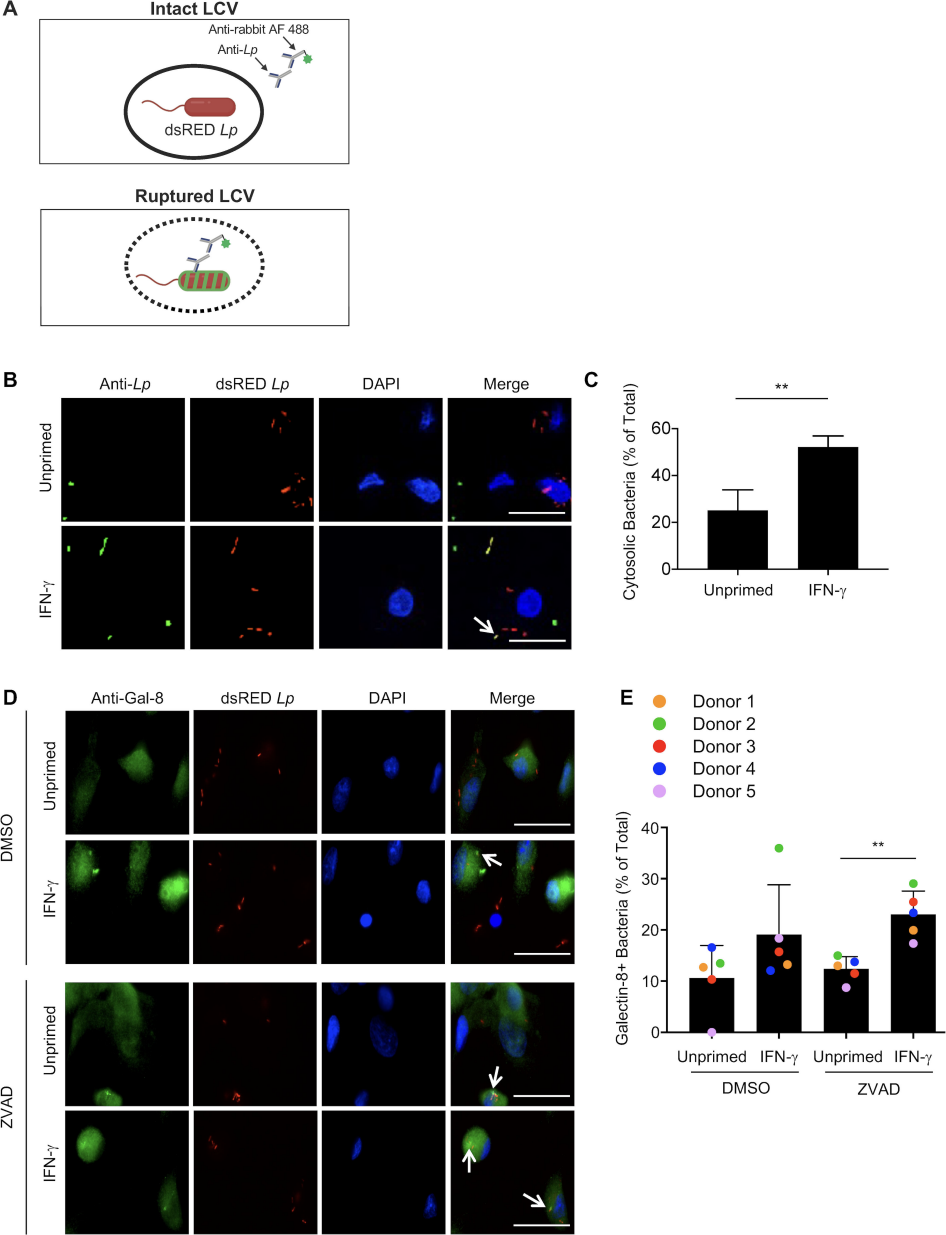
IFN-γ promotes galectin-8 recruitment to LCVs and cytosolic exposure of *L. pneumophila* in hMDMs. (**A**) Schematic of vacuolar *Lp*, which fluoresces red, and cytosolic *Lp*, which is stained green and fluoresces red. (**B, C**) Primary hMDMs were either unprimed or primed with IFN-γ (100 U/mL) overnight and infected with dsRED-expressing T4SS+ *Lp* for 2 h. (**D–E**) Primary hMDMs were either left unprimed or primed with IFN-γ (100 U/mL) overnight, followed by treatment with DMSO or ZVAD 1 h before infection with dsRED-expressing T4SS+ *Lp* for 2 h. (**B**) Representative fluorescence micrographs of anti-*Lp* staining in digitonin-permeabilized cells. Representative of three independent experiments using hMDMs from different healthy donors. (**C**) Quantification of the percentage of hMDMs harboring cytosolic *Lp* out of total infected hMDMs. Graphs show the mean and s.d. of technical triplicates and are representative of three independent experiments using hMDMs from different healthy human donors. **P < 0.01 by unpaired *t*-test. (**D**) Representative fluorescence micrographs of cells stained for Gal-8. Representative of five independent experiments using hMDMs from different healthy human donors. (**E**) Quantification of the percentage of hMDMs containing Gal-8+ *Lp* out of total infected hMDMs. Shown are the pooled results of five independent experiments using hMDMs from different healthy human donors. Each data point represents the mean of triplicate infected wells from an individual donor. **P < 0.01 by paired *t*-test. (**B, D**) Scale bars, 20 µM. White arrows indicate anti-*Lp-* or anti-Gal-8-stained *L. pneumophila*.

We next wanted to determine how IFN-γ and GBP1 promote inflammasome responses to *L. pneumophila*. Thus, we tested whether IFN-γ priming leads to increased rupture of LCVs, which would allow *L. pneumophila* to become more accessible for cytosolic recognition by inflammasome sensors. We first utilized a differential permeabilization assay to distinguish between vacuolar and cytosolic *L. pneumophila* (20, 53). We infected unprimed and IFN-γ-primed hMDMs with dsRED-expressing T4SS+ *Lp* and then treated with the detergent digitonin, which selectively permeabilizes the plasma membrane while leaving intracellular membranes intact. The cells were then immunostained with an antibody for *L. pneumophila*, followed by staining with an Alexa 488-labeled secondary antibody that fluoresces green. Thus, dsRED-expressing *L. pneumophila* contained within an intact vacuole only fluoresce red, while dsRED-expressing *L. pneumophila* within a ruptured vacuole will fluoresce both green and red (Fig. 6A). Around 25% of unprimed hMDMs contained *L. pneumophila* exposed to the cytosol, whereas a significantly increased percentage of IFN-γ-primed hMDMs, more than 50%, contained *L. pneumophila* exposed to the cytosol, as they stained with anti-*L*. *pneumophila* antibody and fluoresced green (Fig. 6B and C). Treatment with the detergent saponin, which permeabilizes all cell membranes, prior to staining resulted in similar percentages of unprimed and IFN-γ-primed hMDMs containing bacteria that were stained by anti-*L*. *pneumophila* antibody (Fig. S8A and B). The secondary antibody stained only in the presence of anti-*L*. *pneumophila* antibody (Fig. S8C), indicating that the secondary antibody does not directly stain *L. pneumophila*. To confirm that digitonin only permeabilized the plasma membrane and not intracellular membranes in hMDMs, we also stained digitonin-permeabilized or saponin-permeabilized cells with either an anti-calnexin antibody or an anti-protein disulfide isomerase (PDI) antibody (53). Calnexin is an integral ER membrane protein and PDI is an enzyme located within the ER lumen; therefore, digitonin treatment should only permeabilize the host cell plasma membrane and lead to anti-calnexin staining but not anti-PDI staining. In contrast, there should be both anti-calnexin and anti-PDI staining in saponin-permeabilized cells since saponin permeabilizes the host cell plasma membrane as well as intracellular membranes. We observed anti-calnexin staining in both digitonin-treated and saponin-treated hMDMs (Fig. S9A), while we detected anti-PDI staining in only saponin-treated hMDMs and not in digitonin-treated hMDMs, indicating that the differential permeabilization assay functions properly (Fig. S9B). Thus, these data indicate that IFN-γ priming of hMDMs promotes the rupture of the LCV and increased access of *L. pneumophila* to the host cell cytosol.

We next used an additional assay to monitor LCV damage. We stained unprimed or IFN-γ-primed hMDMs with galectin-8 (Gal-8), a known vacuolar lysis marker that binds to ruptured pathogen vacuoles (20). Furthermore, the hMDMs were pretreated with either DMSO or ZVAD to determine whether caspase activity contributes to LCV rupture. We found that IFN-γ priming significantly increased the percentage of Gal-8+ *L. pneumophila*-infected hMDMs (Fig. 6D and E). Secondary antibody alone had minimal background staining (Fig. S10A). Furthermore, we observed a similar increase in the percentage of Gal-8+ *L. pneumophila*-infected hMDMs regardless of DMSO or ZVAD pretreatment, suggesting that LCV rupture is independent of caspase activity. We also observed that a similar percentage of IFN-γ-primed THP-1 cells contained Gal-8+ *L. pneumophila*, regardless of DMSO or ZVAD treatment (Fig. S10B and C). Secondary antibody alone had negligible background staining (Fig. S10D). Overall, these data indicate that IFN-γ promotes the rupture of the LCV independently of caspase activity, resulting in increased *L. pneumophila* exposure to the host cell cytosol.

**Fig 7:**
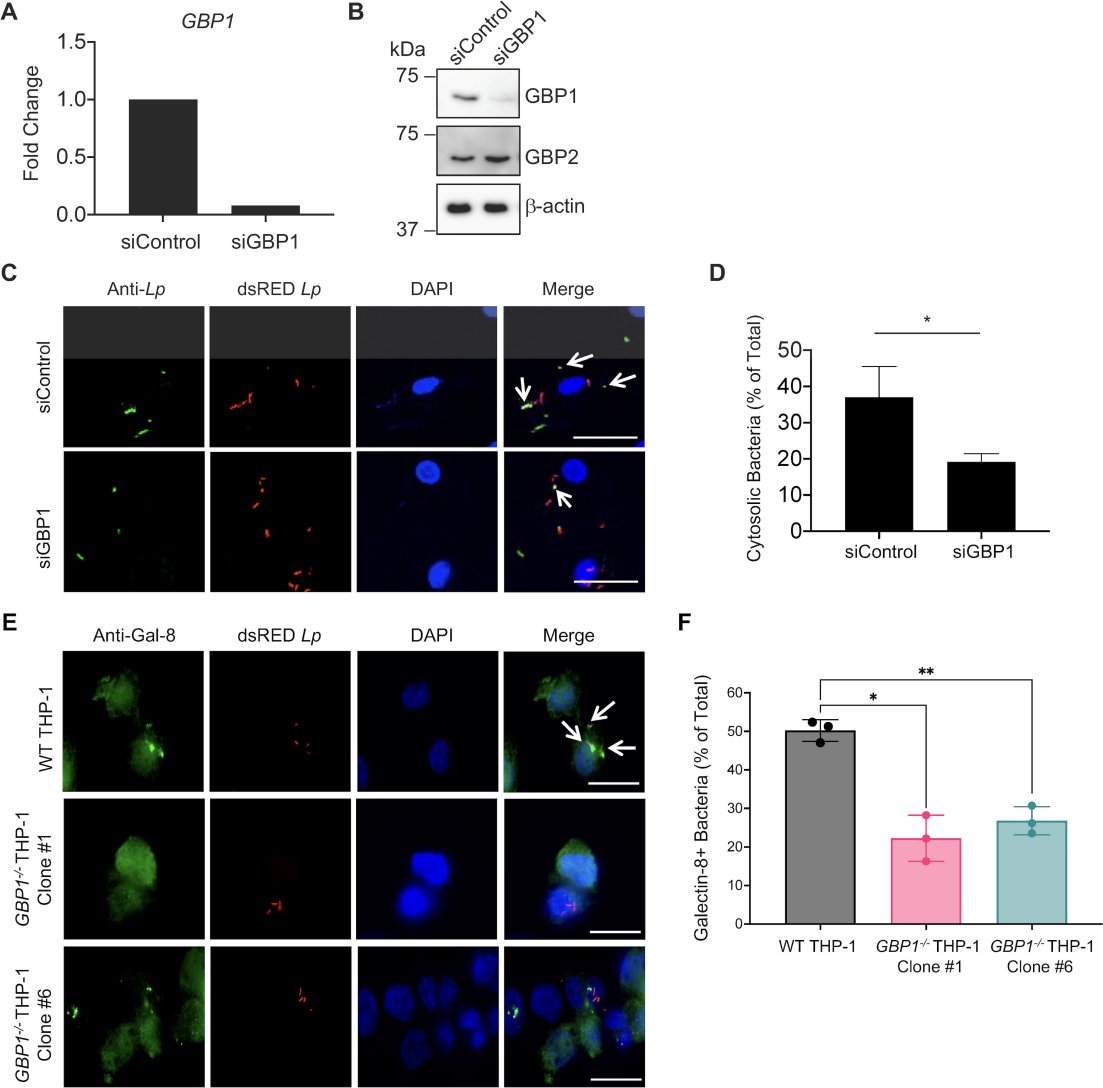
GBP1 promotes galectin-8 colocalization with the LCV and cytosolic exposure of *L. pneumophila* in IFN-γ-primed macrophages. (**A–D**) Primary hMDMs were transfected with *GBP1* siRNA (siGBP1) or scrambled control siRNA (siControl) for at least 48 h, primed with IFN-γ (100 U/mL) overnight, and infected with dsRED-expressing T4SS+ *Lp* for 2 h. (**E, F**) PMA-differentiated WT THP-1 cells and *GBP1^−/^* ^−^ clones were primed with IFN-γ (100 u/mL) overnight and infected with dsRED-expressing T4SS+ *Lp* for 1 h. (**A**) *GBP1* levels in *GBP1* siRNA-treated mock-infected samples were determined by quantitative RT-PCR. Fold change was calculated by normalizing to the housekeeping gene *HPRT* and then to the siControl sample. (**B**) Immunoblot analysis of mock-infected lysates for GBP1, GBP2, and β-actin. (**C**) Representative fluorescence micrographs of anti-*Lp* staining in digitonin-permeabilized hMDMs. (**D**) Percentage of hMDMs harboring cytosolic *Lp* out of total infected hMDMs. Graph shows the mean and s.d. of technical triplicates and are representative of three independent experiments using hMDMs from different healthy human donors. *P < 0.05 by unpaired *t*-test. (**A–D**) Representative of three independent experiments using hMDMs from different healthy human donors. (**E**) Representative fluorescence micrographs of anti-Gal-8 staining in digitonin-permeabilized cells. Representative of three independent experiments. (**F**) Percentage of THP-1 cells harboring Gal-8+ *Lp* out of total infected cells. Shown are the pooled results of three independent experiments. Each data point represents the mean of triplicate infected wells from an individual experiment. *P < 0.05 by unpaired *t*-test and **P < 0.01 by paired *t*-test. (**C, E**) Scale bars, 20 µM. White arrows indicate anti-*Lp*- or anti-Gal-8-stained *L. pneumophila*.

Our data indicate that IFN-γ signaling promotes the rupture of the LCV and that GBP1 colocalizes with and promotes maximal inflammasome responses to *L. pneumophila*. Intriguingly, previous studies show that GBP1 is required for the rupture of the *Toxoplasma-*containing vacuole, whereas it is dispensable for the rupture of the *Salmonella-*containing vacuole (34). Thus, we next asked whether GBP1 contributes to the disruption of the LCV. We first conducted the phagosome integrity assay in *GBP1-*silenced hMDMs primed with IFN-γ. We observed efficient and specific *GBP1* knockdown at the mRNA and protein levels (Fig. 7A and B) and a significantly lower percentage of infected hMDMs containing GBP1+ *L. pneumophila* in the *GBP1* siRNA-treated hMDMs compared to control siRNA-treated hMDMs (Fig. S11A and B). Interestingly, we observed a significant decrease in the percentage of *GBP1* siRNA-treated hMDMs containing anti-*L*. *pneumophila-*stained bacteria following digitonin permeabilization compared to hMDMs treated with control siRNA (Fig. 7C and D). In contrast, following saponin permeabilization of all cellular membranes, a similar percentage of control or *GBP1* siRNA-treated hMDMs contained bacteria that stained positive for anti-*L*. *pneumophila* antibody (Fig. S11D and E), whereas secondary antibody staining alone revealed negligible background staining (Fig. S11C and F). These differential permeabilization data suggest either that GBP1 promotes the rupture of the LCV or, alternatively, that GBP1 promotes anti-*L*. *pneumophila* antibody staining of bacteria within LCVs that are damaged by other IFN-dependent factors.

To distinguish between these two possibilities, we next asked whether GBP1 is required for Gal-8 co-localization with the LCV, as Gal-8 is recruited to damaged vacuoles. We infected IFN-γ-primed WT and *GBP1^−/^* ^−^ THP-1 cells with *L. pneumophila* and stained for Gal-8. Interestingly, we observed that both *GBP1^−/^* ^−^ THP-1 clones had a significant decrease in the percentage of cells harboring Gal-8 +*L. pneumophila* compared to WT THP-1 cells (Fig. 7E and F). Secondary antibody staining alone had minimal background signal (Fig. S11G). Since Gal-8 is recruited to damaged vacuoles, these results, together with the phagosome integrity assay, indicate that GBP1 facilitates the vacuolar rupture of the LCV and increased exposure of *L .pneumophila* to the host cell cytosol in IFN-γ-primed macrophages.

## DISCUSSION

Our data reveal that IFN-γ and human GBP1 promote robust caspase-1- and caspase-4-dependent inflammasome responses to *L. pneumophila* in human macrophages. We find that GBP1 colocalizes with *L. pneumophila* and/or the LCV in a T4SS-dependent manner. Furthermore, our data indicate that IFN-γ and GBP1 facilitate LCV rupture and increased exposure of *L. pneumophila* to the host cell cytosol. Our findings suggest a model in which GBP1 and possibly other IFN-γ-induced components facilitate LCV rupture and the liberation of *L. pneumophila* components into the host cell cytosol to allow for increased inflammasome sensing and activation.

Although mice encode 11 GBPs and humans encode 7 GBPs, there are some GBPs shared between mice and humans, with murine Gbp2 and Gbp5 thought to be the orthologs of human GBP1 and GBP5, respectively, (28). Murine GBPs colocalize with bacterial pathogens that harbor specialized secretion systems that translocate bacterial products into host cells (24, 35). Murine Gbp2 promotes lysis of the SCV and activation of the noncanonical inflammasome (20), whereas other murine GBPs do not mediate vacuole disruption for other bacterial pathogens and instead facilitate lysis of *L. pneumophila* and other bacteria in the cytosol (18, 21). In contrast, human GBP1 colocalizes with *S*. Typhimurium and does not promote disruption of the SCV but still promotes caspase-4-mediated pyroptosis (32, 34). Human GBP1 forms a coat around the cytosolic bacterium *Shigella flexneri* and directly binds bacterial LPS to promote disruption of the bacterial outer membrane (29, 30, 47). Interestingly, human GBP1 promotes the rupture of vacuoles containing the eukaryotic parasite *Toxoplasma gondii* as well as the rupture of *Toxoplasma* membranes (34). Whether human GBP1 also promotes lysis of bacteria-containing vacuoles was unknown. Importantly, our findings reveal that GBP1 targets *L. pneumophila* and/or the LCV in a T4SS-dependent manner and, furthermore, that GBP1 promotes vacuolar disruption and increased exposure of *L. pneumophila* to the host cell cytosol. Thus, human and murine GBP orthologs may have overlapping and distinct functions depending on the pathogen.

Our data show that human GBP1 and GBP2 colocalize with *L. pneumophila* in a T4SS-dependent manner, but whether and how these GBPs are recruited and bind to the LCV and/or bacteria remains to be determined. Human GBP1 associates with damaged endosomes and lysosomes in the absence of infection (35), although the mechanism underlying how GBP1 binds ruptured host membranes is unclear. Murine Gbp2 colocalizes with vacuoles containing bacterial pathogens with virulence-associated secretion systems in a galectin-3-dependent manner (35). Our data showed increased Gal-8 co-localization with LCVs in IFN-γ-primed cells. Whether galectins facilitate human GBP1 recruitment to pathogen-containing vacuoles is unknown and warrants future studies. Furthermore, human and murine GBP1, GBP2, and GBP5 have a C-terminal CaaX prenylation motif that facilitates membrane binding and oligomerization with other GBPs (54). Human GBP1 colocalization with *S. flexneri* and *S*. Typhimurium requires its isoprenylation and GTPase activity (29, 30, 34). It would be of interest to determine whether the CaaX motif in human GBP1 and GBP2 are necessary for colocalization with *L. pneumophila* and/or the LCV and what bacterial or vacuolar components they are binding to. In addition, human GBP1 binds directly to bacterial LPS, and LPS O-antigen enables GBP1 targeting to bacteria (29). Whether LPS O-antigen promotes GBP1 co-localization with *L. pneumophila* would be interesting to examine.

GBP2 is a closely related paralog of GBP1. Although we found that GBP2 colocalized with *L. pneumophila* and/or the LCV in human macrophages*,* siRNA-mediated silencing of *GBP2* did not affect inflammasome activation. It is possible that GBP2 is not required for inflammasome responses to *L. pneumophila* or that siRNA-mediated knockdown in primary hMDMs was not efficient enough to reveal a role for GBP2 during infection. Recent studies indicate that GBP2 cannot bind bacteria directly, although both GBP1 and GBP2 can directly bind and aggregate free LPS to promote caspase-4 activation (55). Thus, a role for GBP2 may be revealed in the absence of GBP1 in promoting inflammasome responses to *L. pneumophila*. Further studies will discern between these possibilities. It would also be of interest to determine whether GBP1 may act as an initiator GBP that recruits additional GBPs to *L. pneumophila* and/or the LCV, similar to what has been observed with *S. flexneri* (29, 30, 56), and whether there is a synergistic role for multiple human GBPs in promoting inflammasome responses to *L. pneumophila*.

Inflammasome activation is triggered in response to sensing of bacterial products within the cytosol. Localization of *L. pneumophila* within its ER-derived vacuole would presumably limit the ability of host cells to recognize bacterial components in the cytosol. However, when the integrity of the LCV is compromised, either by host factors or due to the absence of the T4SS effector SdhA required for maintaining vacuolar integrity, *L. pneumophila* becomes more accessible for recognition by host cytosolic sensors (18). We show that IFN-γ priming in primary human macrophages results in an increased frequency of ruptured LCVs, as indicated by increased Gal-8 co-localization with the LCV and the phagosome integrity assay. These data suggest that IFN-inducible host cell factors promote disruption of the LCV. Furthermore, our data indicate that GBP1 is one such factor. While we cannot conclude that GBP1 is directly mediating the rupture of the LCV, our data indicate that GBP1 somehow facilitates LCV rupture, thus increasing exposure of *L. pneumophila* to the host cell cytosol and making the bacteria more vulnerable to inflammasome sensing. GBP1 may also target and promote destabilization of the outer membrane of *L. pneumophila* to enable the release of bacterial components, including LPS, for inflammasome sensing. Indeed, recombinant GBP1 is capable of binding directly to *L. pneumophila* (47). Intriguingly, human GBP1 promotes the rupture of the host-derived vacuole containing the eukaryotic parasite *Toxoplasma gondii* and the rupture of *Toxoplasma* membranes even though *Toxoplasma* does not contain LPS (34). Furthermore, human GBP1 associates with ruptured host vacuoles in the absence of infection (29, 35). Thus, these findings indicate that GBP1 can bind and facilitate the rupture of host- or pathogen-derived membranes through alternative mechanisms not involving LPS binding. Whether GBP1 colocalizes with *L. pneumophila* and/or the LCV through binding to LPS that is exposed due to initial LCV damage caused by T4SS activity or colocalizes with bacteria and/or the LCV through LPS-independent mechanisms is unclear. In addition, it is unknown how GBP1 facilitates the rupture of PCVs and whether distinct or overlapping mechanisms are employed for different pathogens.

Overall, our findings reveal a critical role for IFN-γ and human GBP1 in enhancing human inflammasome responses against *L. pneumophila*. Our study elucidates a key function of IFN-γ and GBP1 in facilitating the disruption of the LCV. These findings provide insight into human cell-autonomous responses to *L. pneumophila* and reveal distinct roles for human GBPs in promoting inflammasome responses to different pathogens.

## MATERIALS AND METHODS

### Cell culture

THP-1 cells (TIB-202; American Type Culture Collection) and *GBP1^−/^* ^−^ THP-1 single cell clones were maintained in RPMI supplemented with 10% (vol/vol) heat-inactivated FBS, 0.05 nM β-mercaptoethanol, 100 IU/mL penicillin, and 100 µg/mL streptomycin at 37°C in a humidified incubator. The day before stimulation, cells were replated in media without antibiotics in a 48-well plate at a concentration of 2 × 10^5^ cells per well or in a 24-well plate at a concentration of 3.5 × 10^5^ cells per well and incubated with phorbol 12-myristate 13-acetate (PMA) for 24 h to allow differentiation into macrophages. Media was replaced with RPMI without serum for infections in 48-well or 24-well plate. For microscopy experiments, cells were plated on glass coverslips in a 24-well plate.

Primary human monocytes from deidentified healthy human donors were obtained from the University of Pennsylvania Human Immunology Core. Monocytes were cultured in RPMI supplemented with 10% (vol/vol) heat-inactivated FBS, 2 mM l-glutamine, 100 IU/mL penicillin, 100 µg/mL streptomycin, and 50 ng/mL recombinant human M-CSF (Gemini Bio Products). Cells were cultured for 4 days in 10 mL of media in 10 cm dishes at 4–5 × 10^5^ cells/mL, followed by the addition of 10 mL of fresh growth media for an additional 2 days for complete differentiation into macrophages. The day before macrophage stimulation, cells were rinsed with cold PBS, gently detached with trypsin-EDTA (0.05%), and replated in media without antibiotics and with 25 ng/mL M-CSF in a 48-well plate at a concentration of 1 × 10^5^ cells per well or in a 24-well plate at a concentration of 2 × 10^5^ cells per well. For microscopy experiments, cells were plated on glass coverslips in a 24-well plate.

### IFN-γ priming of macrophages

In infection experiments, primary human monocyte-derived macrophages (hMDMs), PMA-differentiated WT THP-1 cells, and PMA-differentiated *GBP1^−/^* ^−^ THP-1 single cell clones (#1 and #6) were either unprimed or primed overnight with recombinant human IFN-γ (R&D Systems) at a concentration of 100 U/mL. In dose-response experiments, hMDMs were either unprimed or primed with 0.1, 1, 10, or 100 U/mL of IFN-γ for 20 h.

### Bacterial strains and macrophage infection

All *Legionella pneumophila* strains are derived from the serogroup 1 clinical isolate Philadelphia-1. Where indicated, strains utilized were derived from the Lp02 strain (*rpsL, hsdR, thyA*), which is a thymidine auxotroph. The isogenic Lp02 (*rpsL, hsdR, thyA*) flagellin mutant, Δ*flaA* (T4SS+ *Lp*), and avirulent *dotA* mutant, Lp03 (T4SS− *Lp*) were used to infect PMA-differentiated THP-1 cells and primary hMDMs in the absence of exogenous thymidine to avoid the potentially confounding effect of bacterial replication on inflammasome (38, 39, 57). Δ*flaA* (T4SS+) or Δ*dotA* (T4SS−) *L. pneumophila* strains on the JR32 background (*rpsL, hsdR*) carrying pSW001, which allows for constitutive dsRED expression, were used in immunofluorescence experiments (58, 59). All *L. pneumophila* strains were grown as a stationary patch for 48 h on charcoal yeast extract agar plates at 37°C (60). Bacteria were resuspended in PBS and added to cells at a multiplicity of infection (MOI) of 10 or 50 as indicated. Infected cells were then centrifuged at 290 × *g* for 10 min and incubated at 37°C. For immunofluorescence experiments, primary hMDMs were infected for 2 h and PMA-differentiated THP-1 cells (WT or *GBP1^−/^* ^−^ clones) were infected for 1 h. For infection experiments involving THP-1-derived macrophages (WT or *GBP1^−/^* ^−^ clones), cells were infected for 2 h. For additional infection experiments involving primary hMDMs, cells were infected for 4 h. For all experiments, mock-infected cells were treated with PBS.

### Caspase inhibitor treatments

25 micromolar (µM) of caspase-1 inhibitor Ac-YVAD-cmk (Sigma-Aldrich SML0429) and 20 µM of pan-caspase inhibitor Z-VAD (61)-FMK (SM Biochemicals SMFMK001) were added to primary hMDMs 1 h before infection. DMSO treatment was used as a vehicle control.

### siRNA-mediated knockdown

All of the Silencer Select siRNA oligos targeting human *GBP* mRNAs were purchased from Thermo Fisher Scientific. Individual siRNA targeting *CASP4* (s2412)*, CASP5* (s2417)*, GBP1* (s5620), *GBP2* (s5623), *GBP3* (5628), *GBP4* (s41805), and *GBP5* (s41810) were used. The two Silencer Select negative control siRNAs (Silencer Select Negative Control No. 1 siRNA and Silencer Select Negative Control No. 2 siRNA) were purchased from Life Technologies (Ambion). In experiments where *CASP4* or *CASP5* were individually knocked down or knocked down together as well as in experiments where *GBP1–5* were individually knocked down, primary hMDMs were replated in media without antibiotics in a 48-well plate, as described above, 3 days before infection. Two days before infection, 30 nM of total siRNA were transfected into macrophages using HiPerFect transfection reagent (Qiagen) following the manufacturer’s protocol. Sixteen hours before infection, media was replaced with fresh antibiotic-free media either containing 100 U/mL IFN-γ or left unprimed for *CASP4* and *CASP5* knockdown experiments, while only fresh antibiotic-free media containing 100 U/mL IFN-γ was used for *GBP1–5* knockdown experiments. In immunofluorescence experiments where *GBP1* was knocked down, primary hMDMs were replated in media without antibiotics on glass coverslips in a 24-well plate as described above 4 days before infection. Three days before infection, 5 pmol of total siRNA was transfected into macrophages using Lipofectamine RNAiMAX transfection reagent (Thermo Fisher Scientific) following the manufacturer’s protocol. Sixteen hours before infection, media was replaced with fresh antibiotic-free media containing 100 U/mL IFN-γ.

### Quantitative RT-PCR analysis

RNA was isolated using the RNeasy Plus Mini Kit (Qiagen) following the manufacturer’s protocol. Cells were lysed in 350 µL RLT buffer with β-mercaptoethanol and centrifuged through a QIAshredder spin column (Qiagen) prior to RNA isolation. cDNA was synthesized from isolated RNA using SuperScript II Reverse Transcriptase (Invitrogen) following the manufacturer’s protocol. Quantitative PCR was conducted with the CFX96 real-time system from Bio-Rad using the SsoFast EvaGreen Supermix with Low ROX (Bio-Rad). Transcript levels for each gene were normalized to the housekeeping gene HPRT for each sample, and samples were normalized to unprimed sample or to control siRNA-treated sample using the 2^−ΔΔ*Ct*^ (cycle threshold) method to calculate fold change. Relative expression was calculated by normalizing gene-specific transcript levels to HPRT transcript levels for each sample using the 2^−Δ*Ct*^ method. Primer sequences from PrimerBank (62 – 64) used for *HPRT1*, *GBP1-6*, *CASP4*, and *CASP5* or from Lagrange et al. for *GBP7* are in Table S1.

### LDH cytotoxicity assay

Macrophages were infected in a 48-well plate as described above, and harvested supernatants were assayed for cell death by measuring loss of cellular membrane integrity via lactate dehydrogenase (LDH) activity. LDH release was quantified using an LDH Cytotoxicity Detection Kit (Clontech) according to the manufacturer’s instructions and normalized to mock-infected cells.

### Real-time propidium iodide uptake assay

To measure live kinetics of cell membrane permeability, THP-1 cells were plated as described above in a black, flat-bottom 96-well plate (Cellstar), primed with 100 U/mL IFN-γ for 24 h, and infected with T4SS+ *Lp* at an MOI of 50 in media containing 1× HBSS without phenol red, 20 mM HEPES, and 10% (vol/vol) heat-inactivated FBS. Infected cells were centrifuged at 290 × *g* for 10 min. The cells were supplemented with 5 µM propidium iodide (PI, P3566, Invitrogen) and incubated for 10 min at 37°C to allow the cells to equilibrate. Then, the plate was sealed with adhesive optical plate sealing film (Microseal, Bio-Rad) and placed in a Synergy H1 microplate reader (BioTek) pre-heated to 37°C. PI fluorescence was measured every hour for 4 h.

### ELISA

Macrophages were infected in a 48-well plate as described above, and harvested supernatants were assayed for cytokine levels using ELISA kits for human IL-1β (BD Biosciences) and IL-18 (R&D Systems).

### Immunoblot analysis

In experiments where macrophages were plated in a 48-well plate, cells were lysed in 1× SDS/PAGE sample buffer, and low-volume supernatants (90 µL media per well of a 48-well plate) were mixed 1:1 with 2 × SDS/PAGE sample buffer containing Complete Mini EDTA-free Protease Inhibitor Mixture (Roche). In experiments where primary hMDMs were plated in a 24-well plate and infected with T4SS− *Lp*, T4SS+ *Lp*, or mock infected with PBS, cells were lysed in 1× SDS/PAGE sample buffer, and supernatants were treated with trichloroacetic acid (TCA) overnight at 4°C and centrifuged at maximum speed for 15 min. Precipitated supernatant pellets were washed with ice-cold acetone, centrifuged at maximum speed for 10 min, and resuspended in 1× SDS/PAGE sample buffer. Protein samples were boiled for 5 min, separated by SDS/PAGE on a 12% (vol/vol) acrylamide gel, and transferred to PVDF Immobilon-P membranes (Millipore). Primary antibodies specific for human IL-1β (clone 8516; R&D Systems), caspase-1 (2225S; Cell Signaling), caspase-4 (4450S; Cell Signaling), caspase-5 (D3G4W; 46680S; Cell Signaling), Gasdermin-D (126-138; G7422; Sigma-Aldrich), GBP1 (ab131255, Abcam), GBP2 (sc-271568, Santa Cruz), GBP4 (17746-1-AP, Proteintech), GBP5 (D3A5O, 67798S; Cell Signaling), and β-actin (4967L; Cell Signaling) were used. HRP-conjugated anti-rabbit IgG (7074S; Cell Signaling) and anti-mouse IgG (7076S; Cell Signaling) secondary antibodies were used. For detection, ECL Western Blotting Substrate or SuperSignal West Femto (both from Pierce Thermo Scientific) were used as the HRP substrate.

### Development of CRISPR/Cas9-edited THP-1 cells

To knock out *GBP1* in THP-1 cells, pLentiCRISPR v2 plasmids encoding the specific gRNA and Cas9 were purchased from GenScript. The following target sequence was used: *GBP1* gRNA 3: ACAAAGAGACGATAGCCCCC. Initial production of lentiviral particles used the pCMV-VSV-G and psPAX2 plasmids generously provided by Paul Bates at the University of Pennsylvania. HEK293T cells were plated at 2.0 × 10^6^ cells in a 10 cm-dish in 10 mL of DMEM supplemented with 10% (vol/vol) heat-inactivated FBS, 2 mM l-glutamine, 100 IU/mL penicillin, and 100 µg/mL streptomycin. Twenty-four hours after plating HEK293T cells in a 10 cm dish, plasmids were transfected using the Lipofectamine 2000 protocol. Transfected HEK293T cells were incubated at 37°C for 18 h, followed by removal of the media and replacement with 6 mL of fresh HEK293T cell growth media per dish. Twenty-four hours later, the supernatant containing the lentiviral particles was harvested and filtered using a 0.22-µM filter. 5 × 10^5^ THP-1 cells were infected with 1 mL of supernatant containing lentiviral particles treated with 8 µg/mL of polybrene and plated in a TC-treated 12-well plate. Infected THP-1 cells were then centrifuged at 1,250 × *g* for 90 min at 25°C. Cells were then pipetted out of the wells and added to a conical tube for each gRNA condition. Cells were centrifuged at 805 × *g* for 3 min at 25°C. Media was aspirated, and cells were resuspended in fresh THP-1 growth media. Two milliliters of resuspended cells was added per well of a 12-well plate. Cells were incubated at 37°C for 48 h. After 48 h, puromycin was added at a final concentration of 1 µg/mL. Cells were maintained in puromycin for 2–3 weeks and then harvested for western blot analysis and clonal selection. For clonal selection, cells were plated at a concentration of 0.5 cell per 200 µL of THP-1 growth media in flat-bottom 96-well plates and incubated at 37°C for 4–6 weeks until outgrowth of single cell clones was noticeable. Twelve single cell clones for each gene were expanded. The cells from each single cell clone were plated in a 48-well plate at a concentration of 2 × 10^5^ cells per well in 500 µL of media treated with PMA for differentiation into macrophages, the next day either stimulated with IFN-γ overnight or left unstimulated, and harvested for DNA, RNA, and western blot analyses. The single cell clones were then validated as indicated below.

### Validation of CRISPR/Cas9 Knockout THP-1 Single Cell Clones for GBP1

After selecting three *GBP1^−/^* ^−^ single cell clones based on the absence of *GBP1* mRNA and protein expression, DNA from the single cell clones was purified using the DNeasy Blood and Tissue Kit (Qiagen). The genomic region comprising the gRNA target sequence for each gene was amplified by PCR using the following primers (all 5′ → 3′):

*GBP1* forward: GGTGAGGAGGCTGTCAGTTTC

*GBP1* reverse: ACTCTCTTTGATGAGCACCTAGGAC

The PCR product was purified using the QIAquick PCR purification Kit (Qiagen). The purified PCR product was then ligated into the pGEM-T vector (Promega) and transformed into DH5α high-efficiency competent cells. Following blue-white screening, colony PCR screening was conducted on 10–15 white colonies using the primer sequences shown above. Purified plasmids from positive colonies were sequenced using the M13/pUC primer: 5′ CCCAGTCACGACGTTGTAAAACG 3′.

### Immunofluorescence microscopy

Primary hMDMs or THP-1 cells were plated on glass coverslips in a 24-well plate as described above. After 2 h of infection in primary hMDMs or 1 h of infection in THP-1 cells with dsRED-*Lp*, cells were washed two times with PBS and fixed with 4% paraformaldehyde for 10 min at 37°C. Following fixation, cells were washed and permeabilized with 0.2% Triton X-100 for 10 min. Cells were washed, blocked with 10% BSA for 1 h, and stained with anti-GBP1 or anti-GBP2 primary antibodies for 1 h at RT or overnight at 4°C with anti-galectin-8 primary antibody. Cells were washed with PBS and incubated with the appropriate Alexa-Fluor-conjugated secondary antibodies for 1 h followed by additional washes and mounted on glass slides with DAPI mounting medium (Sigma Fluoroshield).

For anti-GBP1 and anti-GBP2 antibody staining, primary antibodies used were rabbit anti-GBP1 (1:100 dilution; ab131255; Abcam) and mouse anti-GBP2 (1:50 dilution; sc-271568; Santa Cruz). Secondary antibodies were goat anti-rabbit conjugated to Alexa Fluor 488 (4412S; Cell Signaling) and goat anti-mouse conjugated to Alexa Fluor 488 (A11029; Life Technologies) and used at a dilution of 1:4,000. Coverslips were imaged on a Leica SP5 FLIM confocal microscope at 63× magnification, and the percentage of infected cells containing GBP1^+^ or GBP2^+^ intracellular bacteria out of the total number of infected cells was quantified.

For galectin-8 antibody staining experiments, goat anti-galectin-8 (1:200 dilution in primary hMDMs and 1:100 in THP-1 cells; AF1305; R&D Systems) and donkey anti-goat IgG conjugated to Alexa Fluor 488 (1:4,000 dilution; A11055; Invitrogen). Coverslips were imaged on an inverted fluorescence microscope (IX81; Olympus) at a magnification of 60×, and the percentage of infected cells containing galectin-8^+^ LCVs out of the total number of infected cells was quantified.

### Phagosome integrity assay

The phagosome integrity assay was performed as previously published (53), with some modifications. To confirm that the phagosome integrity assay functions properly in hMDMs, we stained digitonin-treated and saponin-treated cells with anti-calnexin antibody (1:100; ADI-SPA-860-D; Enzo Life Sciences) or anti-PDI antibody (1:100; ADI-SPA-890-D; Enzo Life Sciences), followed by anti-rabbit Alexa Fluor 594 (1:4,000; A-11012; Invitrogen). Primary hMDMs were plated on glass coverslips in a 24-well plate as described above. Cells were washed three times with KHM buffer (110 mM potassium acetate, 20 mM HEPES, and 2 mM MgCl_2_, pH 7.3) and incubated for 1 min with 50 µg/mL digitonin (Sigma-Aldrich) or 0.1% saponin (Sigma-Aldrich) in KHM buffer. Cells were washed three times with KHM buffer and stained for 15 min at 37°C with primary antibody to calnexin or PDI in KHM buffer with 3% BSA. Cells were washed with PBS, fixed, and quenched with 0.1 M glycine for 10 min. Cells were washed and incubated with secondary antibody anti-rabbit Alexa Fluor 594 for 1 h, followed by washes and mounted on glass slides with DAPI mounting medium. Cells were analyzed by microscopy using an inverted fluorescence microscope (IX81; Olympus) at a magnification of 60×.

To distinguish between cytosolic and vacuolar bacteria, primary hMDMs were plated on glass coverslips in a 24-well plate as described above and infected with dsRED-*Lp*. After 2 h of infection, cells were washed three times with KHM buffer and incubated for 1 min in KHM buffer with 50 µg/mL digitonin. Cells were washed three times with KHM buffer and stained for 15 min at 37°C with rabbit anti-*L*. *pneumophila* antibody (1:1,000 dilution; gift from Craig Roy) in KHM buffer with 3% BSA. Cells were washed with PBS, fixed, and quenched with 0.1 M glycine for 10 min. Cells were washed and incubated with secondary antibody anti-rabbit Alexa Fluor 488 (1:4,000 dilution; 4412S; Cell Signaling) for 1 h, followed by washes and mounted on glass slides with DAPI mounting medium. Cells were analyzed by microscopy. 0.1% saponin in KHM buffer was used as a positive control for this assay. Coverslips were imaged on a Leica SP5 FLIM confocal microscope at a magnification of 63×, and the percentage of infected cells harboring cytosolic bacteria out of the total number of infected cells was quantified.

### Statistical analysis

GraphPad Prism software was used for graphing of data and all statistical analyses. Statistical significance for experiments with THP-1 cells was determined using the unpaired two-way Student’s *t-*test. Statistical significance for hMDMs was determined using the paired two-way *t-*test in experiments comparing multiple donors and the unpaired two-way *t-*test in experiments involving infections with dsRED-expressing *Lp* for immunofluorescence assay. In hMDM experiments that compare cells from multiple donors, data were graphed so that each data point represents the mean of triplicate wells for each donor, and all statistical analysis was conducted comparing the means of each experiment. Differences were considered statistically significant if the *P* value was <0.05.

## SUPPLEMENTAL MATERIAL

Supplemental figures mbio.01707-23-s0001.pdf Figures S1 to S11.

Supplemental legends and table mbio.01707-23-s0002.docx Legends for Figures S1 to S11; Table S1.

ASM does not own the copyrights to Supplemental Material that may be linked to, or accessed through, an article. The authors have granted ASM a non-exclusive, world-wide license to publish the Supplemental Material files. Please contact the corresponding author directly for reuse.
