# Supplementary material for: Human GBP1 facilitates the rupture of the Legionella-containing vacuole and inflammasome activation
Source: mBio. 2023 Sep 22;14(5):e01707-23. doi: 10.1128/mbio.01707-23 (PMC10653807; doi:10.1128/mbio.01707-23)
Supplement: Supplemental figures — Figures S1 to S11. [file mbio.01707-23-s0001.pdf]

Supplemental Figures

Human GBP1 promotes rupture of the *Legionella*-containing vacuole and inflammasome activation

Antonia R. Bass[a,b], Marisa S. Egan[a], Jasmine Alexander-Floyd[a,c,*], Natasha Lopes Fischer[a,d,*], Jessica Doerner[a,e], Sunny Shin[a#]

[a]Department of Microbiology, Perelman School of Medicine, University of Pennsylvania, Philadelphia, PA 19104

Current Address:

[b]MRL, Merck & Co., Inc., Rahway, NJ

[c]Center for Breakthrough Medicines, King of Prussia, PA

[d]Regeneron, Tarrytown, NY

[e]Bristol Myers Squibb

*JAF and NLF contributed equally to this work.

#Address correspondence to Sunny Shin, sunshin@pennmedicine.upenn.edu.

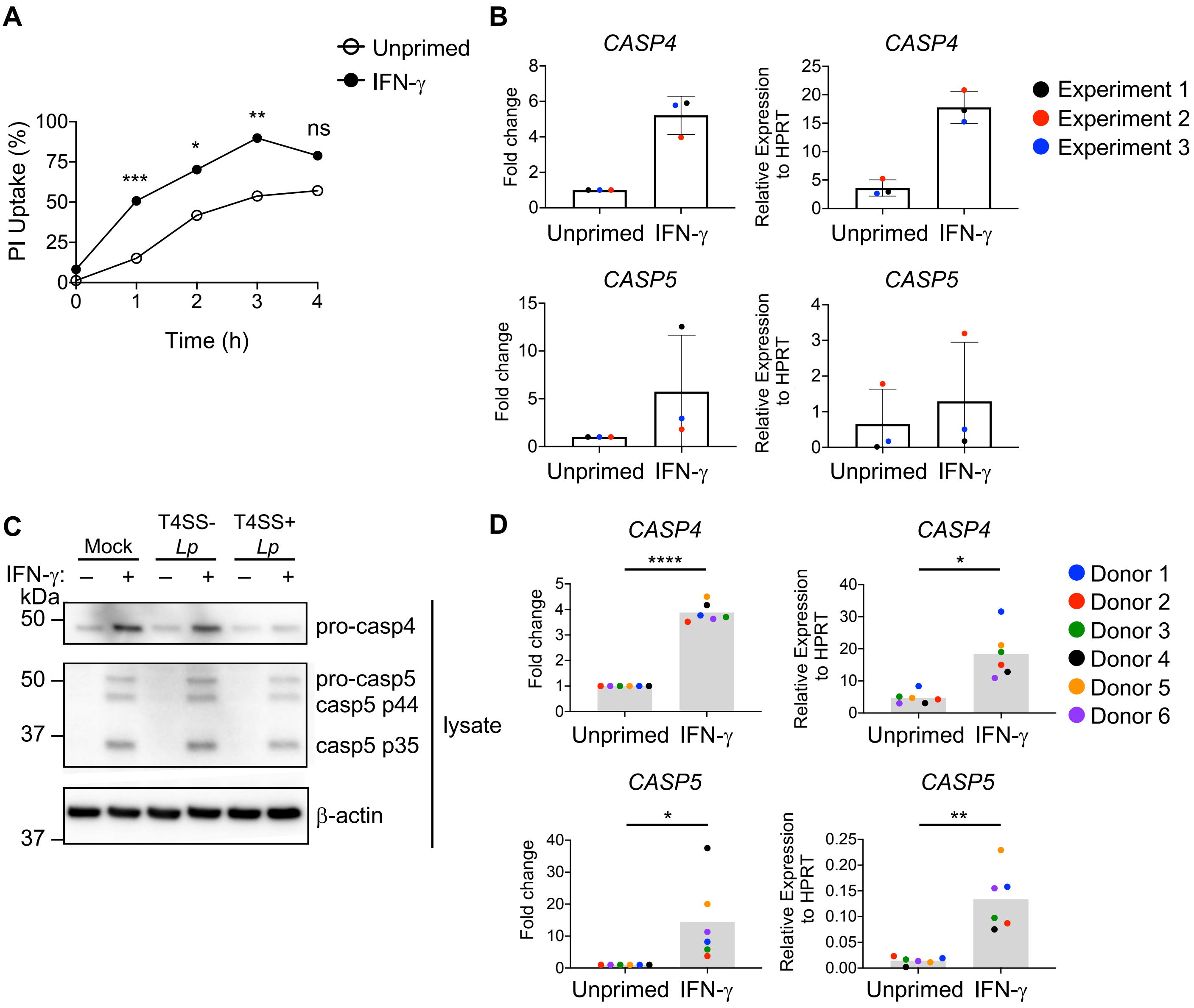
A
Unprimed
IFN-γ
PI Uptake (%)
Time (h)
***
*
**
ns
B
CASP4
CASP5
Fold change
Relative Expression to HPRT
Unprimed
IFN-γ
Experiment 1
Experiment 2
Experiment 3
C
Mock
T4SS- Lp
T4SS+ Lp
IFN-γ:
kDa
pro-casp4
pro-casp5
casp5 p44
casp5 p35
β-actin
lysate
D
CASP4
CASP5
****
*
**
Donor 1
Donor 2
Donor 3
Donor 4
Donor 5
Donor 6

Figure S1

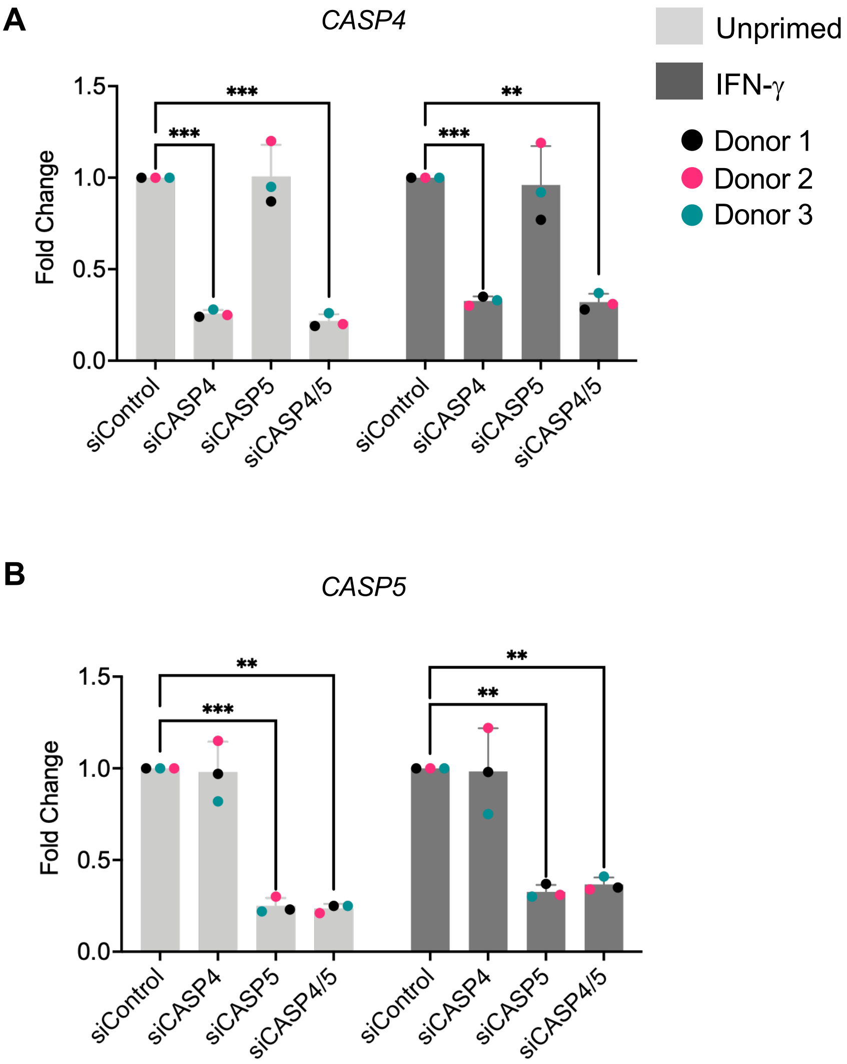
A
CASP4
Unprimed
IFN-γ
Donor 1
Donor 2
Donor 3
Fold Change
1.5
1.0
0.5
0.0
***
***
**
***
siControl
siCASP4
siCASP5
siCASP4/5
B
CASP5
**
***
**
**

Figure S2

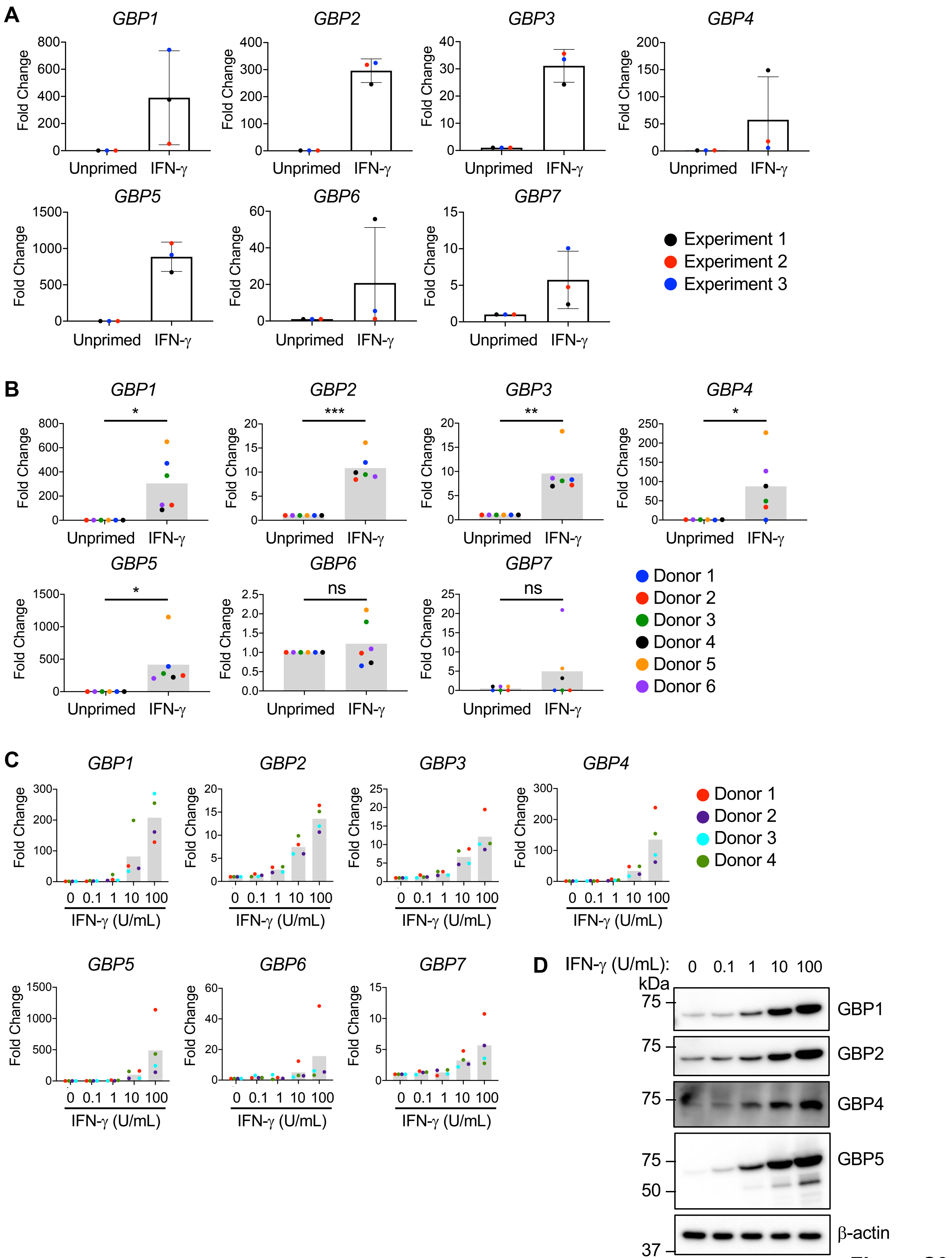
A
GBP1
GBP2
GBP3
GBP4
GBP5
GBP6
GBP7
Fold Change
Unprimed
IFN-γ
Experiment 1
Experiment 2
Experiment 3
B
*
***
**
ns
Donor 1
Donor 2
Donor 3
Donor 4
Donor 5
Donor 6
C
IFN-γ (U/mL)
0 0.1 1 10 100
D
IFN-γ (U/mL): 0 0.1 1 10 100
kDa
75
50
37
GBP1
GBP2
GBP4
GBP5
β-actin

Figure S3

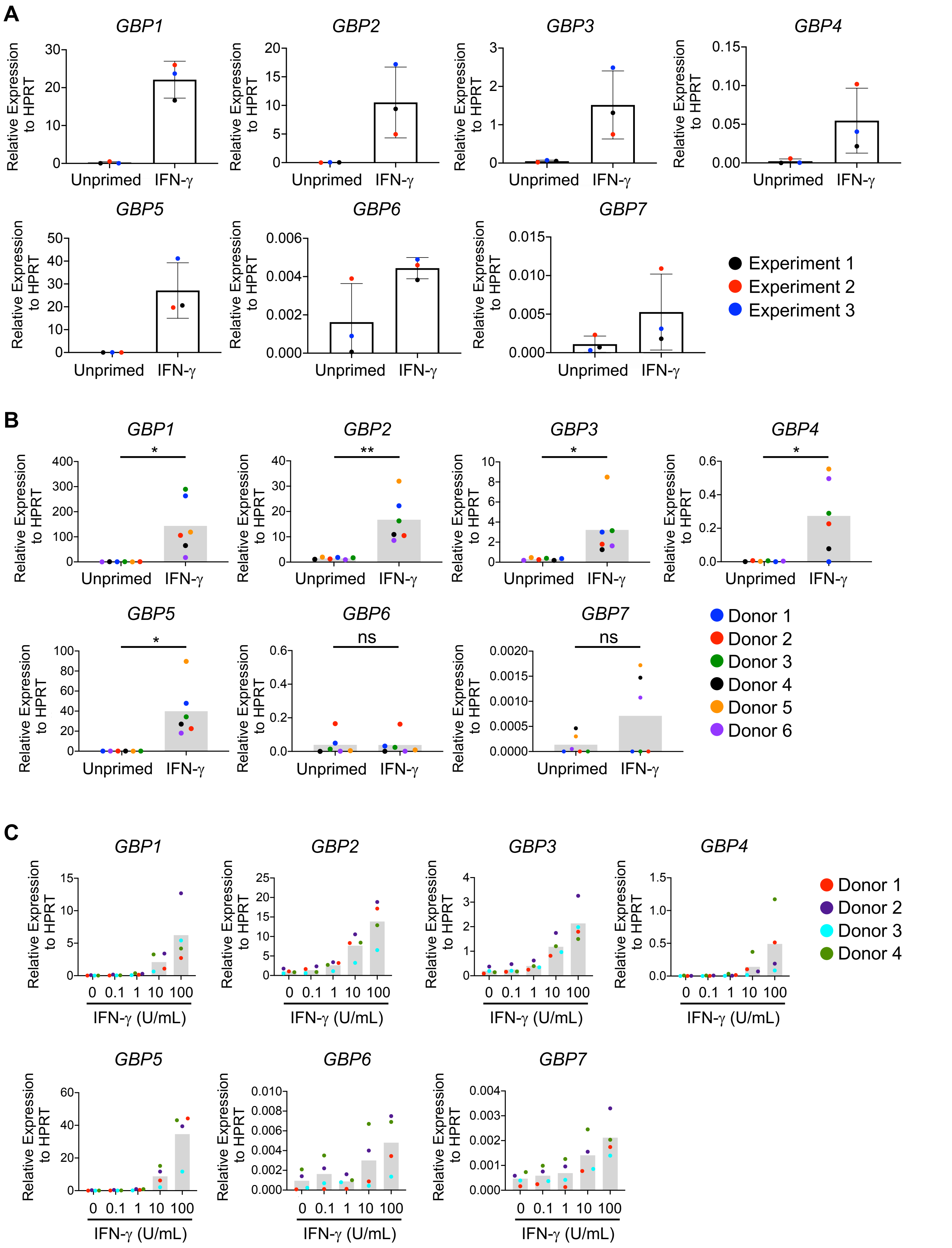
A
GBP1
GBP2
GBP3
GBP4
GBP5
GBP6
GBP7
Relative Expression to HPRT
Unprimed
IFN-γ
Experiment 1
Experiment 2
Experiment 3
B
*
**
ns
Donor 1
Donor 2
Donor 3
Donor 4
Donor 5
Donor 6
C
0
0.1
1
10
100
IFN-γ (U/mL)

Figure S4

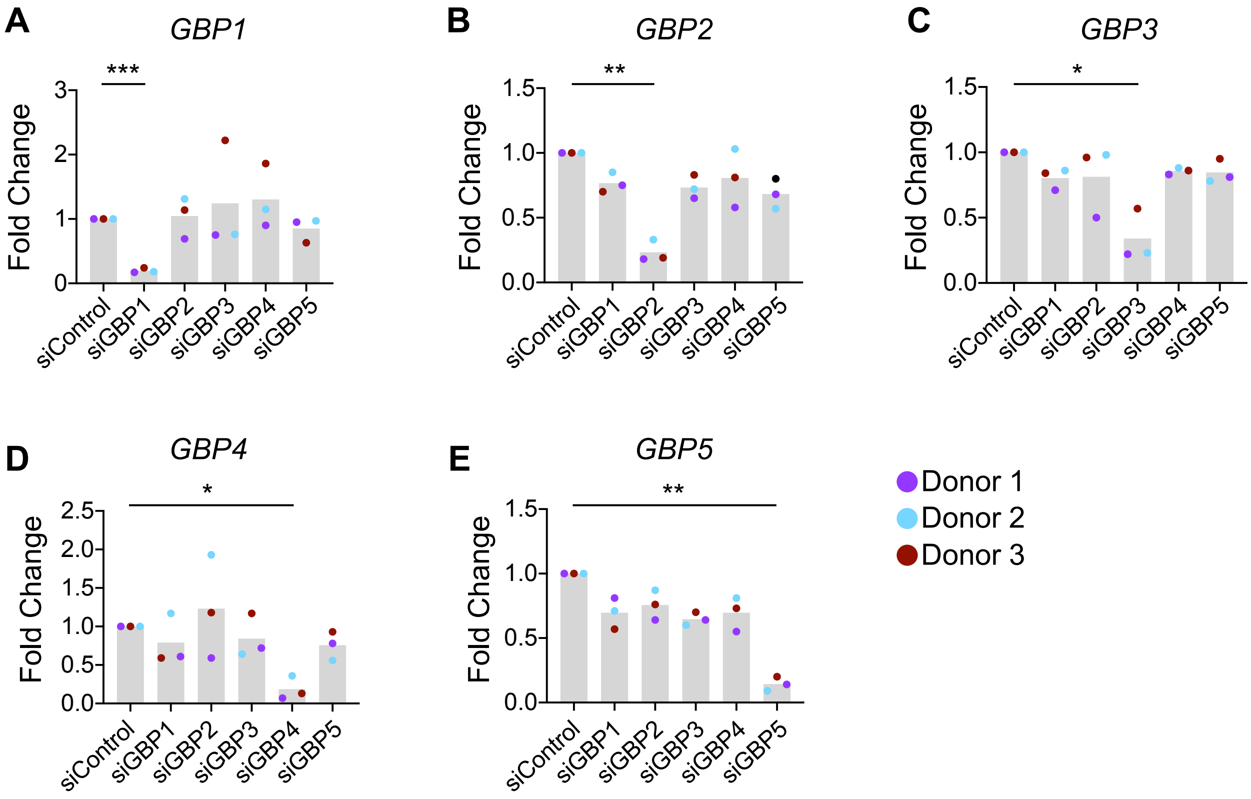
A
GBP1
***
B
GBP2
**
C
GBP3
*
D
GBP4
*
E
GBP5
**
Fold Change
siControl
siGBP1
siGBP2
siGBP3
siGBP4
siGBP5
Donor 1
Donor 2
Donor 3

Figure S5

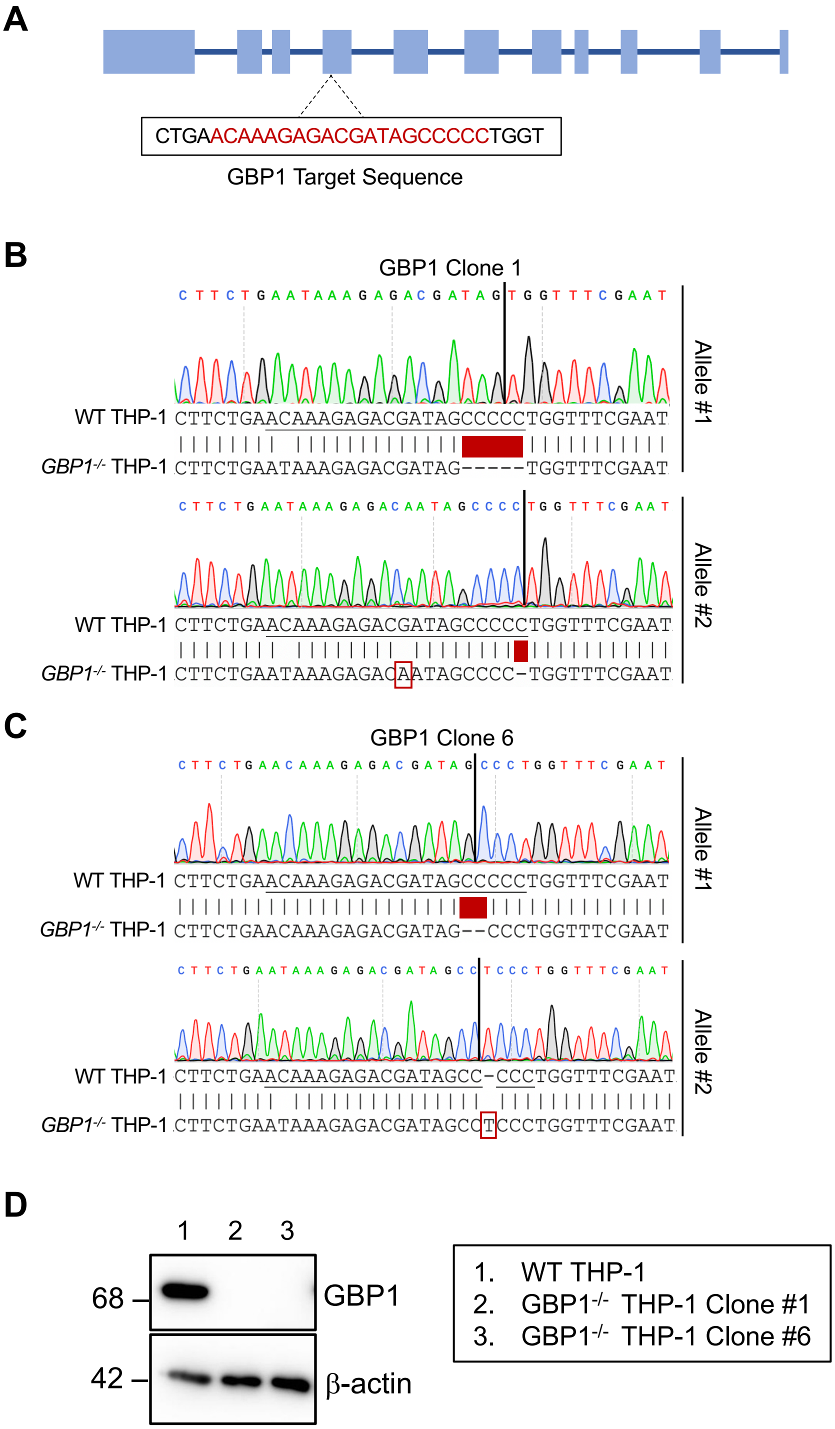
A
CTGAACAAAGAGACGATAGCCCCCTGGT
GBP1 Target Sequence
B
GBP1 Clone 1
Allele #1
WT THP-1 CTTCTGAACAAAGAGACGATAGCCCCCTGGTTTCGAAT
GBP1-/- THP-1 CTTCTGAATAAAGAGACGATAG-----TGGTTTCGAAT
Allele #2
WT THP-1 CTTCTGAACAAAGAGACGATAGCCCCCTGGTTTCGAAT
GBP1-/- THP-1 CTTCTGAATAAAGAGACAATAGCCCC-TGGTTTCGAAT
C
GBP1 Clone 6
Allele #1
WT THP-1 CTTCTGAACAAAGAGACGATAGCCCCCTGGTTTCGAAT
GBP1-/- THP-1 CTTCTGAACAAAGAGACGATAG--CCCTGGTTTCGAAT
Allele #2
WT THP-1 CTTCTGAACAAAGAGACGATAGCC-CCCTGGTTTCGAAT
GBP1-/- THP-1 CTTCTGAATAAAGAGACGATAGCCTCCCTGGTTTCGAAT
D
1 2 3
68
42
GBP1
β-actin
1. WT THP-1
2. GBP1-/- THP-1 Clone #1
3. GBP1-/- THP-1 Clone #6

Figure S6

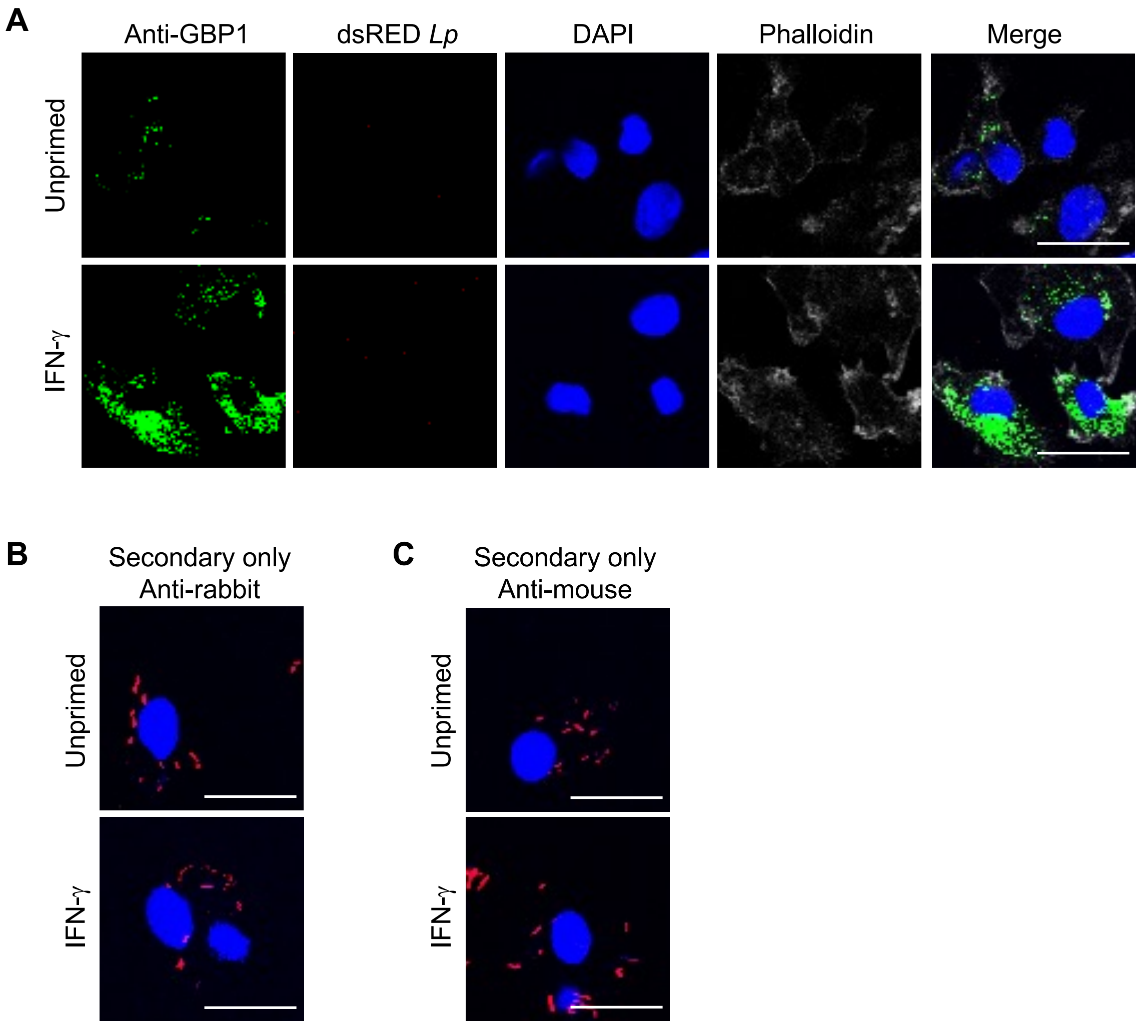
A
Anti-GBP1
dsRED Lp
DAPI
Phalloidin
Merge
Unprimed
IFN-γ
B
Secondary only
Anti-rabbit
Unprimed
IFN-γ
C
Secondary only
Anti-mouse
Unprimed
IFN-γ

Figure S7

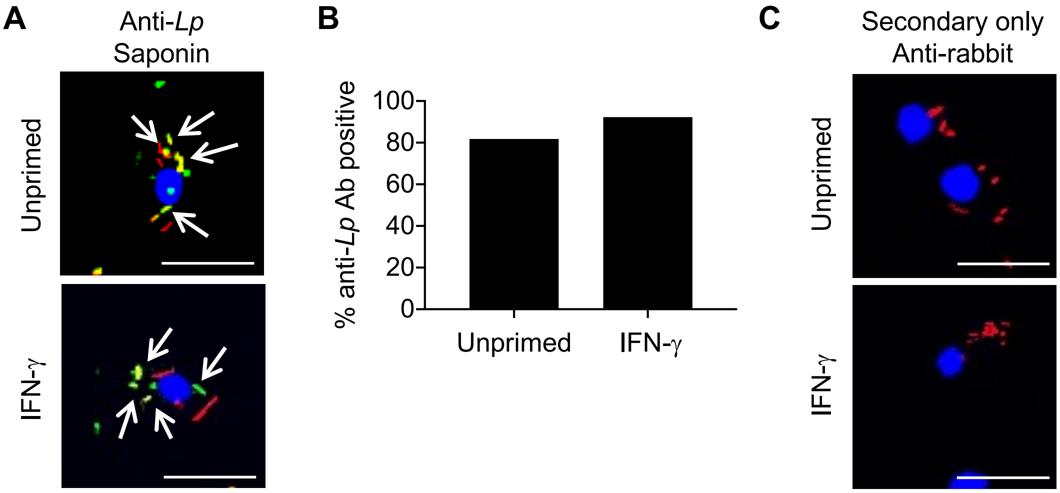
A
Anti-*Lp*
Saponin
Unprimed
IFN-γ
B
% anti-*Lp* Ab positive
100
80
60
40
20
0
Unprimed
IFN-γ
C
Secondary only
Anti-rabbit
Unprimed
IFN-γ

Figure S8

A

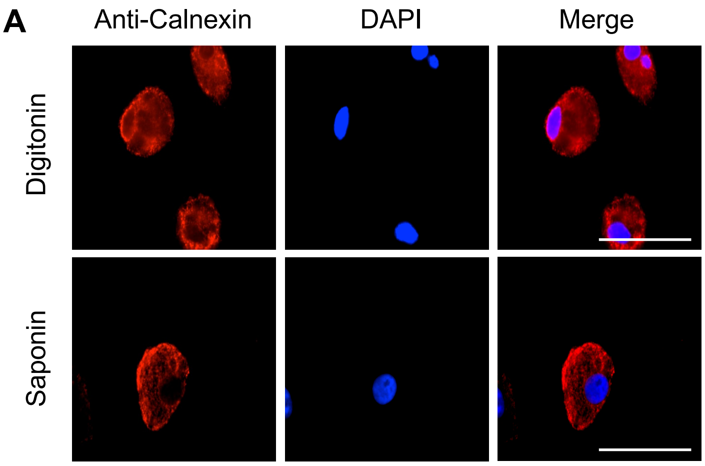
Anti-Calnexin
DAPI
Merge
Digitonin
Saponin

B

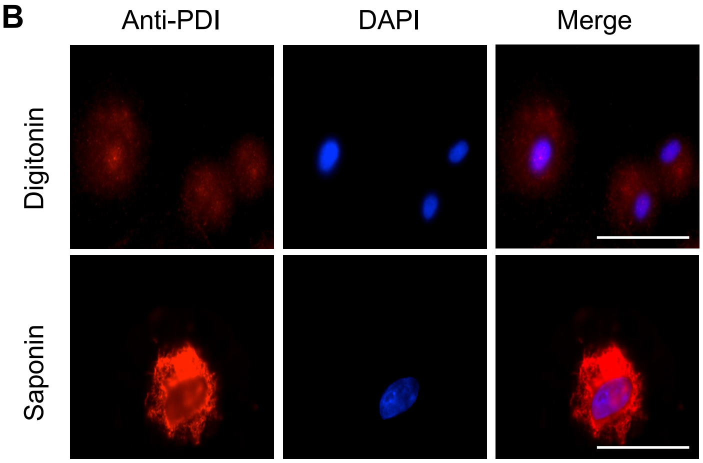
Anti-PDI
DAPI
Merge
Digitonin
Saponin

Figure S9

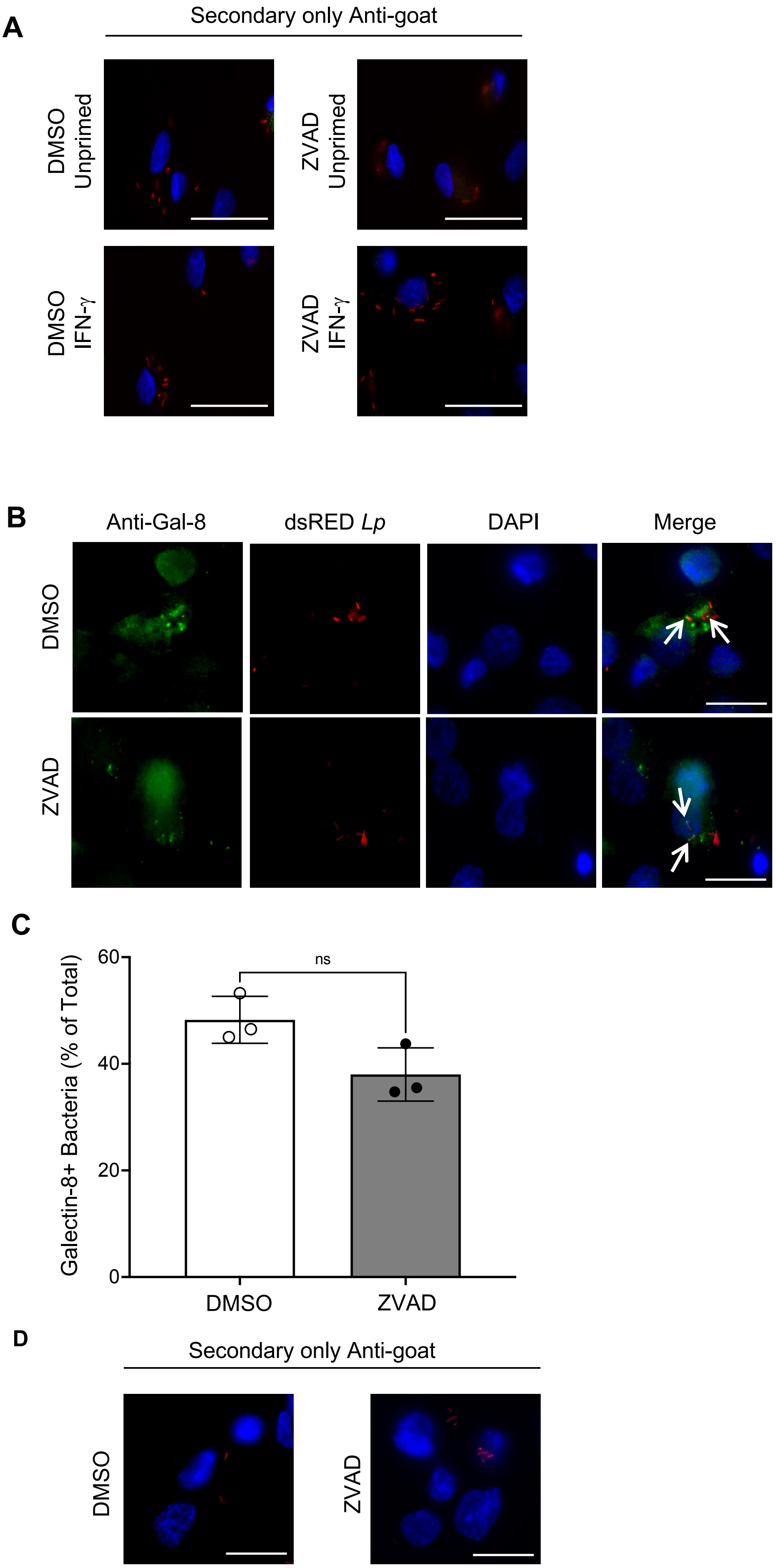
A
Secondary only Anti-goat
DMSO Unprimed
ZVAD Unprimed
DMSO IFN-γ
ZVAD IFN-γ
B
Anti-Gal-8
dsRED Lp
DAPI
Merge
DMSO
ZVAD
C
Galectin-8+ Bacteria (% of Total)
60
40
20
0
ns
DMSO
ZVAD
D
Secondary only Anti-goat
DMSO
ZVAD

Figure S10

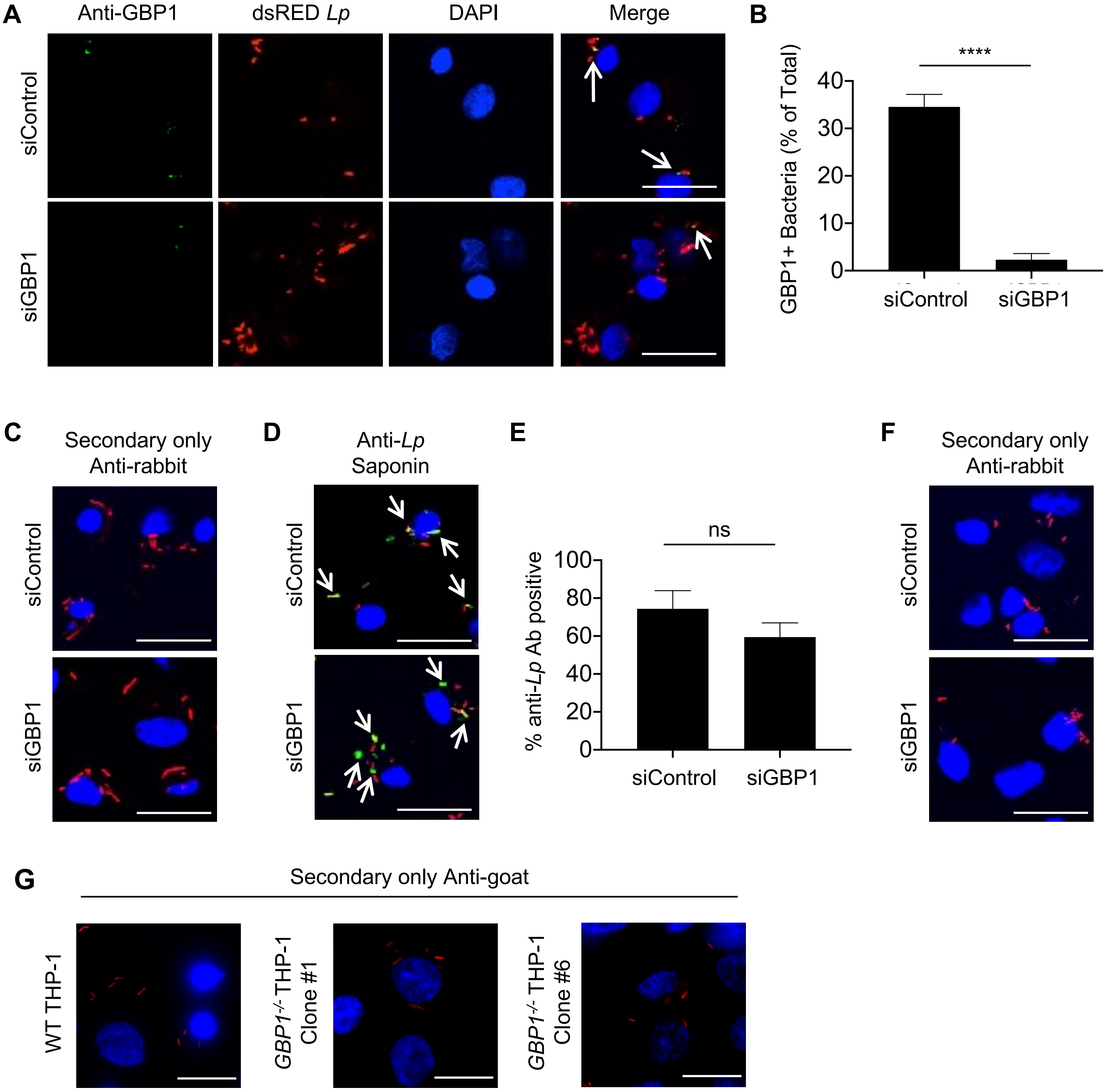
A
Anti-GBP1
dsRED Lp
DAPI
Merge
siControl
siGBP1
B
****
GBP1+ Bacteria (% of Total)
40
30
20
10
0
siControl
siGBP1
C
Secondary only
Anti-rabbit
siControl
siGBP1
D
Anti-Lp
Saponin
siControl
siGBP1
E
ns
% anti-Lp Ab positive
100
80
60
40
20
0
siControl
siGBP1
F
Secondary only
Anti-rabbit
siControl
siGBP1
G
Secondary only Anti-goat
WT THP-1
GBP1-/- THP-1
Clone #1
GBP1-/- THP-1
Clone #6

Figure S11
