# Supplementary material for: Human GBP1 facilitates the rupture of the Legionella-containing vacuole and inflammasome activation
Source: mBio. 2023 Sep 22;14(5):e01707-23. doi: 10.1128/mbio.01707-23 (PMC10653807; doi:10.1128/mbio.01707-23)
Supplement: Supplemental legends and table — Legends for Figures S1 to S11; Table S1. [file mbio.01707-23-s0002.docx]

**Supplemental material**

**Fig. S1.** IFN-γ promotes inflammasome responses to *L. pneumophila* and caspase-4 and caspase-5 expression in human macrophages. (A) Time course of PI uptake in PMA-differentiated THP-1 cells that were either unprimed or primed with IFN-γ (100 U/ml) for 24 h and infected with T4SS+ *Lp* MOI=50*.* Data are representative of three independent experiments with each data point representing the mean of triplicate infected wells. *p< 0.05, **p< 0.01, ***p< 0.001 by unpaired t-test. PMA-differentiated THP-1 cells (B) or primary hMDMs (D) were either unprimed or primed with IFN-γ (100 U/mL) for 18 or 20 hours, respectively. Transcript levels of *CASP4* and *CASP5* were determined by qRT-PCR. Fold change was calculated by normalizing to the housekeeping gene HPRT for each sample and then to the unprimed sample. Relative expression of each gene was calculated by normalizing to the housekeeping gene HPRT. Shown are pooled results of three independent experiments (B) or six independent experiments using hMDMs from different healthy human donors (D), with each data point representing the value for each experiment (B) or an individual donor (D). *p< 0.05, **p< 0.01, and ****p< 0.0001 by paired t-test. (C) PMA-differentiated THP-1 cells were either unprimed or primed with IFN-γ (100 U/ml) overnight and infected with T4SS- *Lp*, T4SS+ *Lp*, or mock-infected with PBS for two hours. Immunoblot analysis was performed on PMA-differentiated THP-1 lysates for full-length caspase-4 (pro-casp4), full-length caspase-5 (pro-casp5), caspase-5 intermediates (casp5 p44 and casp5 p35), and β-actin (β-actin blot from same experiment also shown in Fig. 1C). Representative of three independent experiments.

**Fig. S2.** siRNA-mediated silencing of *CASP4* and *CASP5* in hMDMs. (A and B) Primary hMDMs were transfected with 30 nM of scrambled control siRNA (siControl), or siRNAs targeting *CASP4* (siCASP4), *CASP5* (siCASP5), or both (siCASP4/5). After 28 hours of siRNA treatment, cells were either unprimed or primed with IFN-γ (100 U/mL) overnight and mock-infected with PBS for four hours. Transcript levels of *CASP4* (A) and *CASP5* (B) were determined by qRT-PCR. Fold change was calculated by normalizing to the housekeeping gene HPRT and then to the siControl sample for either unprimed or IFN-γ-primed samples. Shown are the pooled results of three independent experiments using hMDMs from different healthy human donors. Each data point represents an individual donor. **p< 0.01 and ***p< 0.001 by paired t-test.

**Fig. S3.** Expression of human GBPs is induced by IFN-γ. PMA-differentiated THP-1 cells (A) or primary hMDMs (B) were either unprimed or primed with IFN-γ (100 u/mL) for 18 or 20 hours, respectively. (C and D) hMDMs were unprimed or primed with IFN-γ at the indicated concentrations for 20 hours. (A-C) Transcript levels of *GBP1-7* were determined by qRT-PCR. Fold change was calculated by normalizing to the housekeeping gene HPRT for each sample and then to the unprimed sample. Shown are the pooled results of three independent experiments (A) or six independent experiments using hMDMs different healthy human donors (B), with each data point representing an individual experiment (A) or an individual donor (B). *p< 0.05, **p< 0.01, and ***p< 0.001 by paired t-test. (C) Shown are the pooled results of four independent experiments using hMDMs from different healthy human donors. Each data point represents an individual donor. (D) Immunoblot analysis for GBP1, GBP2, GBP4, GBP5, and β-actin. Representative of four independent experiments using hMDMs from different healthy human donors.

**Fig. S4.** Human GBPs are transcriptionally upregulated by IFN-γ in macrophages. PMA-differentiated THP-1 cells (A) or primary hMDMs (B) were either unprimed or primed with IFN-γ (100 u/mL) for 18 or 20 hours, respectively. (C) hMDMs were unprimed or primed with IFN-γ at the indicated concentrations for 20 hours. (A-C) Transcript levels of *GBP1-7* were determined by qRT-PCR and relative expression of each gene was calculated by normalizing to the housekeeping gene HPRT. Shown are the pooled results of three independent experiments (A) or six independent experiments using hMDMs from different healthy human donors (B), with each data point representing the value for each experiment (A) or an individual donor (B). *p< 0.05 and **p< 0.01 by paired t-test. (C) Shown are the pooled results of four independent experiments using hMDMs from different healthy human donors. Each data point represents the value of an individual donor.

**Fig. S5.** siRNA-mediated silencing of human *GBP1-5* in hMDMs. (A-E) Primary hMDMs were transfected with 30nM siRNA specific for each GBP or scrambled control siRNA (siControl), primed with IFN-γ (100 U/mL) overnight, and mock-infected with PBS. Transcript levels of *GBP1* (A), *GBP2* (B), *GBP3* (C), *GBP4* (D), and *GBP5* (E) in mock-infected samples treated with individual *GBP-*specific siRNAs were determined by qRT-PCR. Fold change was calculated by normalizing to the housekeeping gene HPRT and then to the siControl sample. Shown are the pooled results of three independent experiments using hMDMs from different healthy human donors. Each data point represents an individual donor. *p< 0.05, **p< 0.01, and ***p< 0.001 by paired t-test.

**Fig. S6.** Validation of CRISPR/Cas9-generated *GBP1^-/-^* THP-1 single cell clones. (A) Schematic representation of the *GBP1* gene with exons (filled boxes) and introns (lines). The guide RNA target sequence for *GBP1* is highlighted in red. (B and C) Shown are the mutations of the two alleles for *GBP1^-/-^* clone 1 (B) and *GBP1^-/-^* clone 6 (C) by electropherogram and sequence alignment with WT THP-1 genomic DNA. The GBP1 target sequence is underlined, and nucleotide deletions are indicated by the red filled-in boxes. Nucleotide insertion or switch is marked by a red outline. The gRNA target sequence is indicated in the electropherogram by a black line. (D) PMA-differentiated WT THP-1 cells and *GBP1^-/-^* clones were primed with IFN-γ (100 U/mL) for 18 h. Immunoblot analysis of lysates for GBP1 and β-actin.

**Fig. S7.** GBP1 is distributed throughout the cytoplasm in uninfected hMDMs. (A-C) Primary hMDMs were either unprimed or primed with IFN-γ (100 U/mL) overnight and infected with dsRED-expressing T4SS+ *Lp* or left uninfected for two hours. (A) Representative fluorescence micrographs of anti-GBP1 staining in uninfected hMDMs. (B and C) Representative fluorescence micrographs of dsRED-T4SS+ *Lp-*infected hMDMs stained with Alexa Fluor 488-conjugated anti-rabbit (B) or anti-mouse (C) secondary antibodies alone. (A-C) Images are representative of three independent experiments using hMDMs from different healthy human donors. Scale bars, 20 μm.

**Fig. S8.** Controls for phagosome integrity assay. (A-C) Primary hMDMs were either unprimed or primed with IFN-γ (100 U/mL) overnight and infected with dsRED-expressing T4SS+ *Lp* for two hours. (A) Representative fluorescence micrographs of saponin-permeabilized unprimed and IFN-γ-primed dsRED-T4SS+ *Lp-*infected hMDMs stained with anti-*Lp* primary antibody and Alexa Fluor 488-conjugated anti-rabbit secondary antibody. (B) Quantification of the percentage of anti-*Lp* antibody (Ab)-positive hMDMs out of total infected hMDMs. (C) Representative fluorescence micrographs of digitonin-permeabilized unprimed and IFN-γ-primed dsRED-T4SS+ *Lp-*infected hMDMs stained with Alexa Fluor 488-conjugated anti-rabbit secondary antibody alone. (A-C) Data and images are representative of three independent experiments using hMDMs from different healthy human donors. (A and C) Scale bars, 20 μm. White arrows indicate anti-*Lp*-stained *L. pneumophila.*

**Fig. S9.** anti-Calnexin and anti-PDI antibody controls for phagosome integrity assay. (A) Representative fluorescence micrographs of anti-calnexin staining in digitonin-permeabilized or saponin-permeabilized hMDMs. (B) Representative fluorescence micrographs of anti-PDI staining in digitonin-permeabilized or saponin-permeabilized hMDMs. (A-B) Images are representative of three independent experiments using hMDMs from different healthy human donors. Scale bars, 20 μm.

**Fig. S10.** Controls for galectin-8 immunostaining assay and galectin-8 is recruited to the *L. pneumophila-*containing vacuole in IFN-γ-primed THP-1 cells independent of caspase activity. (A) Primary hMDMs were either unprimed or primed with IFN-γ (100 U/mL) overnight, followed by treatment with DMSO or ZVAD one hour before infection with dsRED-expressing T4SS+ *Lp* for two hours. (B-D) PMA-differentiated THP-1 cells were primed with IFN-γ (100 u/mL) overnight, followed by treatment with DMSO or ZVAD one hour before infection with dsRED-expressing T4SS+ *Lp* for one hour. (A) Representative fluorescence micrographs of hMDMs stained with Alexa Fluor 488-conjugated anti-goat secondary antibody alone as a control. Representative of five independent experiments using hMDMs from different healthy human donors. (B) Representative fluorescence micrographs of digitonin-permeabilized cells stained for Gal-8. (C) Percentage of cells harboring Gal-8+ *Lp* out of total infected cells. (D) Representative fluorescence micrographs of IFN-γ-primed dsRED-T4SS+ *Lp*-infected THP-1 cells stained with Alexa Fluor 488-conjugated anti-goat secondary antibody alone as a control. (B and D) Representative of three independent experiments. (C) Shown are the pooled results of three independent experiments. Each data point represents the mean of triplicate infected wells from an individual experiment. ns p> 0.05 by paired t-test. (A, B, D) Scale bars, 20 μm**.** White arrows indicate anti-Gal-8-stained *L. pneumophila.*

**Fig. S11.** Controls for GBP1 immunostaining and phagosome integrity assay. (A-F) Primary hMDMs were transfected with 5 pmol siRNA specific for GBP1 (siGBP1) or scrambled control siRNA (siControl) for at least 48 h, primed with IFN-γ (100 U/mL) overnight, and infected with dsRED-expressing T4SS+ *Lp* for two hours. (G) PMA-differentiated WT THP-1 cells and *GBP1^-/-^* clones were primed with IFN-γ (100 u/mL) overnight, and infected with dsRED-expressing T4SS+ *Lp* for one hour. (A) Representative fluorescence micrographs of anti-GBP1 antibody staining in dsRED-T4SS+ *Lp-*infected hMDMs. (B) Quantification of the percentage of hMDMs containing GBP1+ *Lp* out of total infected hMDMs. (C) Representative fluorescence micrographs of IFN-γ-primed, siControl- and siGBP1-treated dsRED-T4SS+ *Lp*-infected hMDMs stained with only Alexa Fluor 488-conjugated anti-rabbit secondary antibody as a control for GBP1 staining. (D) Representative fluorescence micrographs of anti-*Lp* Ab staining in saponin-permeabilized IFN-γ-primed siControl- and siGBP1-treated dsRED-T4SS+ *Lp*-infected hMDMs and (E) quantification of the percentage of anti-*Lp* Ab positive hMDMs out of total infected hMDMs. (F) Representative fluorescence micrographs of IFN-γ-primed siControl and siGBP1 dsRED-T4SS+ *Lp*-infected hMDMs stained with only Alexa Fluor 488-conjugated anti-rabbit secondary antibody as a control for digitonin phagosome integrity assay. (A-F) Data and images are representative of three independent experiments using hMDMs from different healthy human donors. (G) Representative fluorescence micrographs of cells stained with Alexa Fluor 488-conjugated anti-goat secondary antibody alone as a control. Representative of three independent experiments. (A, C, D, F, G) Scale bars, 20 μm. White arrows indicate anti-GBP1- or anti-*Lp*-stained *L. pneumophila.* ns p> 0.05 and ****p< 0.0001 by unpaired t-test.

**Supplementary Table 1:** qRT-PCR primers

| *HPRT1* forward | CCTGGCGTCGTGATTAGTGAT |
| --- | --- |
| *HPRT1* reverse | AGACGTTCAGTCCTGTCCATAA |
| *GBP1* forward | AGGAGTTCCTTCAAAGATGTGGA |
| *GBP1* reverse | GCAACTGGACCCTGTCGTT |
| *GBP2* forward | CTATCTGCAATTACGCAGCCT |
| *GBP2* reverse | TGTTCTGGCTTCTTGGGATGA |
| *GBP3* forward | ATTCCCTGAAGCTAACGCAAG |
| *GBP3* reverse | GGGCAGATCGAAGACAAAACATT |
| *GBP4* forward | ATGGGTGAGAGAACTCTTCACG |
| *GBP4* reverse | TGCGGTATAGCCCTACAATGG |
| *GBP5* forward | CCATGTGCCTCATCGAGAACT |
| *GBP5* reverse | ACAGGTTGCGTAATGGCAGAC |
| *GBP6* forward | ATGGAATCTGGACCCAAAATGTT |
| *GBP6* reverse | GCTGGTTCACCAATAGCTGCT |
| *GBP7* forward | TGCCTTCTTACCAAGTCCAGA |
| *GBP7* reverse | TCTCTGATGCCATGTTCAGG |
| *CASP4* forward | TCTGCGGAACTGTGCATGATG |
| *CASP4* reverse | TGTGTGATGAAGATAGAGCCCAT |
| *CASP5* forward | TCACCTGCCTGCAAGGAATG |
| *CASP5* reverse | TCTTTTCGTCAACCACAGTGTAG |
